# On the Viability of Carbonyl Hydroboration Catalysed by Aluminium Hydrides

**DOI:** 10.1002/anie.202517007

**Published:** 2025-09-20

**Authors:** Alexa Caise, Barnabé Berger, Aidan Murray, Eugene Kolychev, Jamie Hicks, M. Ángeles Fuentes, Job J. C. Struijs, Jose M. Goicoechea, Simon Aldridge

**Affiliations:** ^1^ Inorganic Chemistry Laboratory Department of Chemistry University of Oxford South Parks Road Oxford OX1 3QR UK; ^2^ Research School of Chemistry The Australian National University Building 137 Sullivans Creek Road Canberra ACT 2601 Australia; ^3^ Department of Chemistry and Center for Research in Sustainable Chemistry (CIQSO) University of Huelva Huelva 21007 Spain; ^4^ Department of Chemistry Indiana University 800 E. Kirkwood Ave Bloomington IN 47405 USA

**Keywords:** Aluminium, Carbonyl hydroboration, Hidden catalysis, Hydride complexes, Pinacolborane

## Abstract

The hydroboration of aldehydes and ketones by pinacolborane (HBpin) catalysed by (Nacnac)^Dipp^Al(OTf)H ((Nacnac)^Dipp^ = HC(MeCDippN)_2_; Dipp = C_6_H_3_ *^i^* Pr_2_‐2,6; Tf = SO_2_CF_3_) was first reported in 2015. This study represented a landmark in main group catalysis, and advanced a widely‐accepted and oft‐cited mechanistic paradigm. However, in contradiction of that study, we show here that: i) the mechanism proposed, involving turnover via Al─O/B─H metathesis at the intermediate (Nacnac)^Dipp^Al(OTf)(OCH_2_Ph), does not occur; ii) when using pre‐purified HBpin, the hydroboration reaction with acetophenone ‘catalysed’ by (Nacnac)^Dipp^Al(OTf)H (reportedly giving 51% conversion over 6 h at 2 mol% loading), actually shows no conversion; and iii) the active species in catalysis is a BH_3_ adduct derived either from the use of impure HBpin, or from the degradation of HBpin by the action of aluminium species present in the reaction mixture. More broadly, our study i) calls into question the nature of the true catalyst species in reports of carbonyl hydroboration by aluminium complexes (since Al─O/B─H metathesis proceeds spontaneously in the *reverse* direction to that necessitated catalytically); and ii) presents further evidence that the hydroboration of benzaldehyde by HBpin is not a good catalytic probe, given the significant rate of the uncatalysed background reaction.

## Graphical abstract

The hydroboration of carbonyl compounds by pinacolborane catalysed by {HC(MeCDippN)_2_}Al(OSO_2_CF_3_)H represented a landmark in main group catalysis, advancing a now widely‐accepted mechanistic paradigm. In contradiction of the original study, we show that: i) turnover via Al─O/B─H metathesis does not occur; ii) when using purified HBpin, the ‘catalysed’ reaction with acetophenone shows no conversion; and iii) the catalytically active species is a BH_3_ adduct.

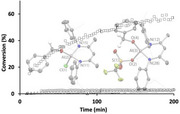

## Introduction

Scientific, economic, and environmental factors have been behind recent efforts to develop alternatives to noble metal catalysts for a range of homogenous processes.^[^ ^1^ ^]^ Within this sphere, approaches based on base metal catalysts^[^ ^2^, ^3^ ^]^ and on frustrated Lewis pairs (FLPs) have seen considerable advances,^[^ ^4^ ^]^ while systems based on *s*‐ and *p*‐block metals have also begun to show promise.^[^ ^5^, ^6^, ^7^ ^]^ Notwithstanding the fact that the first main group metal compound capable of the ambient temperature activation of dihydrogen was reported as late as 2005,^[^ ^8^ ^]^ a number of systems have been reported which show impressive performance in the reduction of unsaturated substrates.^[^ ^9^, ^10^, ^11^, ^12^, ^13^, ^14^, ^15^, ^16^, ^17^, ^18^, ^19^, ^20^, ^21^ ^]^ For example, Jones and coworkers reported a tin amide catalyst that catalyses the hydroboration of ketones with turnover frequencies (>13 300 h^−1^) comparable to those reported for transition metal systems.^[^ ^18^ ^]^

**Scheme 1:**
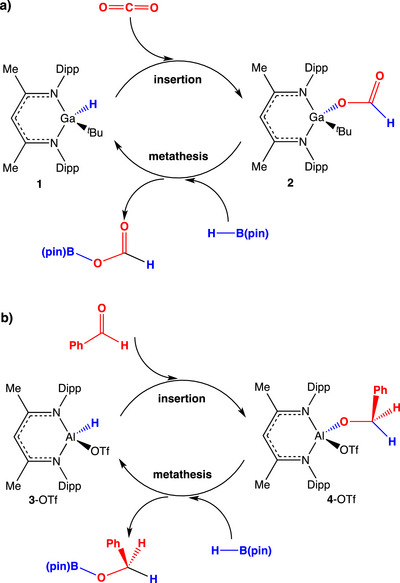
Reported mechanisms for the reduction by pinacolborane a) of CO_2_ catalysed by (Nacnac)^Dipp^Ga(*^t^* Bu)H, and b) of benzaldehyde catalysed by (Nacnac)^Dipp^Al(OTf)H. In both cases turnover is proposed via carbonyl insertion and M─O/B─H metathesis steps.

We have previously shown that gallium β‐diketiminato (‘Nacnac’) complexes can also bring about the catalytic reduction of carbonyl containing substrates including carbon dioxide. (Nacnac)^Dipp^Ga(*^t^* Bu)H (**1**: (Nacnac)^Dipp^ = HC(MeCDippN)_2_; Dipp = 2,6‐*^i^* Pr_2_C_6_H_3_) effects the reduction of CO_2_ by boranes (and silanes), albeit with low turnover frequencies.^[^ ^22^, ^23^ ^]^ In this example, the viability of both mechanistic steps key to catalytic turnover, i.e., CO_2_ insertion and Ga─O/B─H metathesis (Scheme 1a) could be demonstrated via independent stoichiometric reactions, enabled by the isolation of the formate intermediate (Nacnac)^Dipp^Ga(*^t^* Bu)(OCOH) (**2**).^[^ ^22^ ^]^

**Scheme 2:**
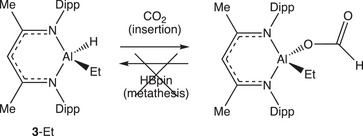
CO_2_ insertion into the Al─H bond of (Nacnac)^Dipp^Al(Et)H (**3**‐Et) to give (Nacnac)^Dipp^Al(Et)(OCOH); lack of reactivity of (Nacnac)^Dipp^Al(Et)(OCOH) towards HBpin.^[^ ^24^ ^].^

The relative cost/terrestrial abundance of gallium and aluminium led us to consider related processes based on the lighter Group 13 metal. In our hands, however, attempts to extend the scope of this chemistry to aluminium‐based catalysts were unsuccessful. For example, while systems such as (Nacnac)^Dipp^Al(Et)H (**3**‐Et) undergo rapid CO_2_ insertion into the more polar Al─H bond (*cf*. Ga─H), the resulting formate derivatives (e.g., (Nacnac)^Dipp^Al(Et)(OCOH)) are resolutely unreactive to B─H containing reagents even at elevated temperatures, and catalytic turnover cannot be achieved in our hands (Scheme 2).^[^ ^24^ ^]^ Superficially, these differences between aluminium and gallium systems are in line with reported E─H/E─O bond strengths for E = B, Al, and Ga (E─H: 377, 282, 260 kJ mol^−1^; E─O: 559, 585, 430 kJ mol^−1^),^[^ ^25^ ^]^ which imply that Ga─O/B─H metathesis should be thermodynamically favourable (albeit marginally so), but that Al─O/B─H metathesis is highly unfavourable.

With this in mind, we have long been intrigued by a landmark (and widely‐cited) report of the catalytic hydroboration of benzaldehyde and acetophenone (among other carbonyl compounds) by (Nacnac)^Dipp^Al(OTf)H (**3**‐OTf), for which a superficially similar Al─O/B─H metathesis process was reported to be a key step in the catalytic cycle (Scheme 1b).^[^ ^26^, ^27^, ^28^, ^29^, ^30^, ^31^, ^32^, ^33^, ^34^, ^35^, ^36^ ^]^ In that study, it was reported that the alkoxide intermediate (Nacnac)^Dipp^Al(OTf)(OCH_2_Ph) (**4**‐OTf), formed by the insertion of PhCHO into the Al─H bond of (Nacnac)^Dipp^Al(OTf)H (**3**‐OTf), reacts with HBpin at room temperature to regenerate **3**‐OTf, and release the product PhCH_2_OBpin. The advantages apparently conferred by the triflate substituent in this chemistry (*cf*. our use of alkyl groups) prompted us to consider its use in a range of reduction processes. During the course of this work, however, we were caused to re‐examine the mechanism previously reported by Yang et al. for the reduction of benzaldehyde catalysed by **3**‐OTf.^[^ ^26^ ^]^ Through isolation and reactivity studies of the proposed intermediate, we find that this chemistry cannot proceed via the mechanism originally proposed, involving Al─O/B─H metathesis. Under the conditions/concentrations originally reported, we find that the reaction of benzaldehyde with pinacolborane occurs to an appreciable extent (ca. 30% over 1 h) even in the absence of a catalyst. The hydroboration of the less reactive substrate acetophenone represents a more suitable probe reaction (and was also investigated by Yang et al.), since it occurs to a negligible degree over 6 h in the absence of a catalyst.^[^ ^26^ ^]^ However, we find that this reaction *does not proceed* in the presence of the ‘catalyst’ **3**‐OTf (at the loading of 2 mol% reported in the original study).^[^ ^26^ ^]^ Catalysis is effected instead by the presence of BH_3_‐containing species in solution, which can arise either as an impurity in the HBpin reagent employed (if not pre‐purified), or generated from HBpin through process(es) mediated by the aluminium hydride ‘catalyst’.

## Results and Discussion

**Figure 1:**
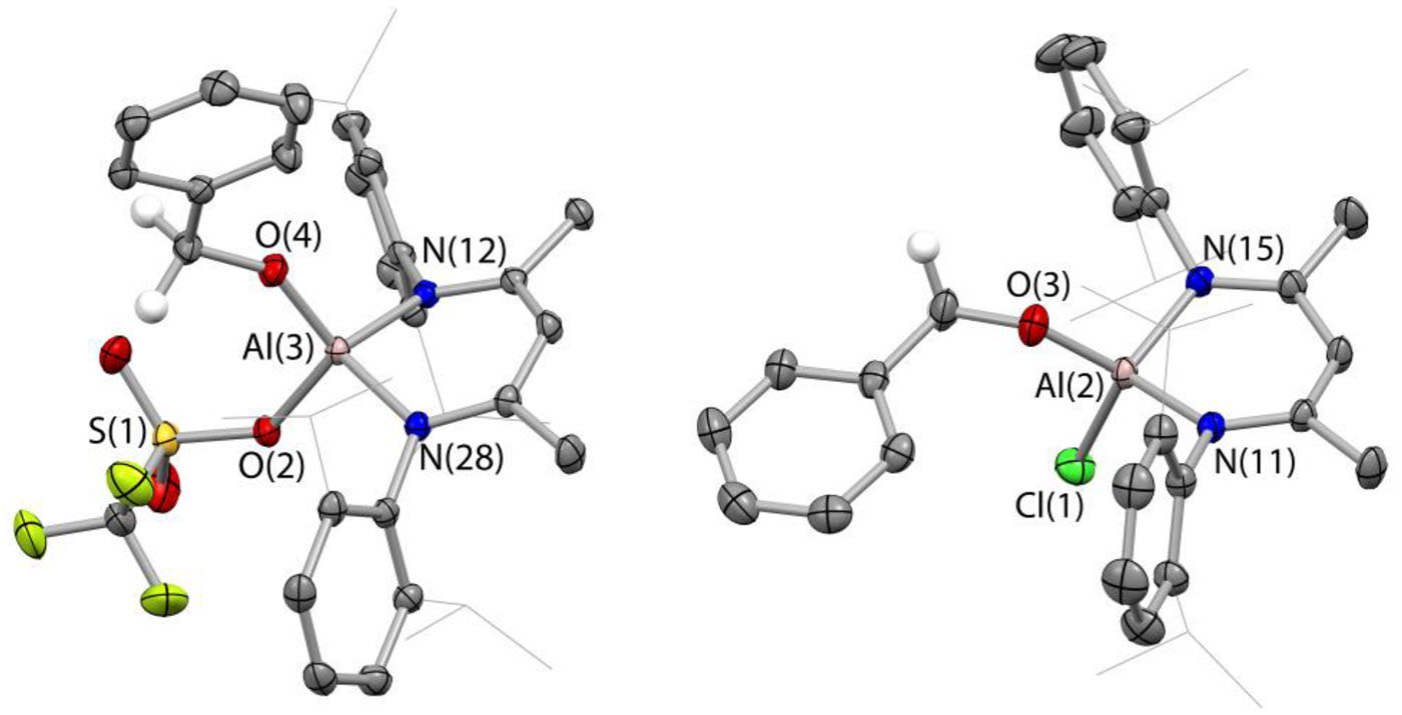
Molecular structures of **4**‐OTf and **4**‐Cl in the solid state as determined by X‐ray crystallography. Most hydrogen atoms omitted and *^i^* Pr substituents shown in wireframe format for clarity; thermal ellipsoids set at the 50% probability level. Key bond lengths (Å) and angles (^o^): (for **4**‐OTf) Al─N 1.859(1), 1.872(1); Al─O(C) 1.677(1); Al─O(S) 1.793(1); N─Al─N 99.0(1); (for **4**‐Cl) Al─N 1.867(1), 1.875(1); Al─O(C) 1.704(1); Al─Cl 2.124(1); N─Al‐─N 99.2(1). For the structure of **4**‐Et see Figure S26.

As a starting point for our study we first sought to obtain isolated samples of the key insertion product (Nacnac)^Dipp^Al(OTf)(OCH_2_Ph) (**4**‐OTf) with a view to probing its chemical competence as an intermediate in the formation of PhCH_2_OBpin via the route outlined in Scheme 1b. Consistent with the report of Yang et al., we find that the reaction of **3**‐OTf with benzaldehyde leads to very rapid formation of **4**‐OTf, with the reaction being complete at room temperature before acquisition of NMR spectra could be achieved (i.e., < 5 min).^[^ ^26^ ^]^ In the absence of previously reported characterising data for **4**‐OTf, we then sought to obtain homogenous samples of this material in order to identify its spectroscopic signatures. **4**‐OTf could be crystallised from pentane solution, and characterised by standard spectroscopic/analytical techniques, together with single crystal X‐ray diffraction (Figure 1). Additionally, the broader scope of this insertion process could be demonstrated: in our hands benzaldehyde will also insert into the corresponding Al─H bonds of (Nacnac)^Dipp^Al(X)H (**3**‐Cl: X = Cl; **3**‐Et: X = Et),^[^ ^24^ ^]^ albeit at significantly slower rates (ca. 1 and 4 h, respectively, for 50% conversion, cf. <5 min for **4**‐OTf) to generate the alkoxides (Nacnac)^Dipp^Al(X)(OCH_2_Ph), **4**‐Cl and **4**‐Et (see Figures 1, S10 and S25). Structurally, **4**‐OTf, **4**‐Cl and **4**‐Et are very similar with near‐identical Al–N distances and N–Al–N angles, although the Al–O distance associated with the OCH_2_Ph group is shorter in the case of **4**‐OTf (1.677(1) vs 1.704(1) and 1.720(1) Å, respectively), consistent with the stronger Al─O bond expected for a more electrophilic aluminium centre.

With structurally authenticated samples of **4**‐OTf in hand we sought to examine the reactivity of this and related systems towards B─H bonds. However, rather than confirming that the reaction with HBpin takes place “at room temperature in C_6_D_6_” over the 1 h period defined by the original catalyst runs,^[^ ^26^ ^]^ we find that no conversion of **4**‐OTf/HBpin into **3**‐OTf and PhCH_2_OBpin occurs even over a period of 14 days under such conditions. The catalytic hydroboration of benzaldehyde by HBpin is reported to take place at room temperature, and this finding caused us to question whether the reaction of **4**‐OTf with HBpin is in fact a viable mechanistic step in this process. We also wanted to test the viability of Al─O/B─H metathesis processes for a wider range of related β‐diketiminate stabilised systems. The reactivity of **4**‐OTf towards the more reactive borane {H(9‐BBN)}_2_, and of **4**‐Cl and **4**‐Et (plus the related compounds (Nacnac)^Dipp^Al(H)(OCH_2_Ph) and (Nacnac)^Dipp^Al(OCH_2_Ph)_2_ previously reported by Masuda and coworkers)^[^ ^37^ ^]^ towards HBpin was therefore examined under comparable conditions. In *none* of these cases was any appreciable conversion to the respective Al─H containing compounds observed either at room temperature or at 60 °C over a period of 5 days. This finding is consistent with our previous studies of β‐diketiminate stabilised aluminum formate compounds, which also show no Al─O/B─H metathesis with boranes.^[^ ^24^ ^]^ Interestingly, however, we find that the related gallane complex (Nacnac)^Dipp^Ga(H)(OCH_2_Ph), formed in a comparable (if much slower) reaction between (Nacnac)^Dipp^GaH_2_ and benzaldehyde (Figure S27), reacts instantly with HBpin to yield PhCH_2_OBpin and reform the gallium dihydride.^[^ ^38^ ^]^

**Scheme 3:**
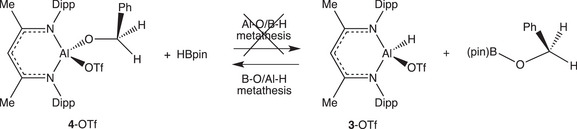
Metathesis chemistry relevant to **3**‐OTf and **4**‐OTf. Our work shows that the reaction proceeds spontaneously in the reverse direction to that proposed in ref 10(a).

Hypothesising that the lack of reactivity of the alane **4**‐OTf towards HBpin might be due either to a prohibitive kinetic barrier under the conditions we had employed, or to more fundamental thermodynamic factors, we decided to examine the viability of the reverse reaction (Scheme 3). Thus, the alkoxyborane PhCH_2_OBpin was synthesised independently from benzyl alcohol and HBpin, and its reactivity towards (Nacnac)^Dipp^Al(H)(OTf) (**3**‐OTf) examined under the conditions employed for the catalytic reaction (C_6_D_6_, room temperature). Informatively, this reaction proceeds spontaneously, leading to quantitative generation of **4**‐OTf and HBpin over a period of ca. 4 h at room temperature (Scheme 3 and Figure S11).^[^ ^39^ ^]^ As such, not only could no evidence be found from our experiments for the key Al─O/B─H metathesis step proposed in Scheme 1b, but its underlying thermodynamic basis is found to be unfavourable under conditions of temperature and solvent comparable to those used in catalytic runs. That the reverse B─O/Al─H metathesis is found to be spontaneous for a wider range of examples can be demonstrated by the fact that the reactions of **3**‐Et, **3**‐Cl, or **3**‐H with PhCH_2_OBpin also proceed at room temperature to yield **4**‐Et, **4**‐Cl, and **4**‐H, respectively.

**Scheme 4:**
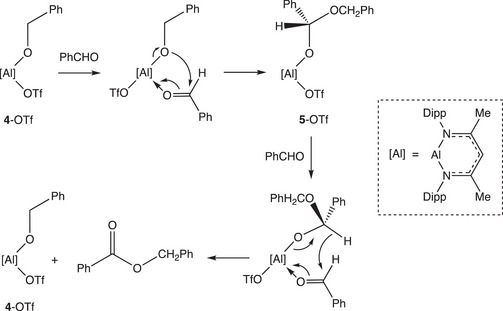
Proposed mechanism for the formation of benzyl benzoate from benzaldehyde via the Tischenko reaction catalysed by **4**‐OTf.^[^ ^40^ ^].^

Having shown that **4**‐OTf (and related systems) do not react with HBpin, we wondered whether turnover might be brought about by an alternative onward step involving reaction with a second equivalent of benzaldehyde. Accordingly, the reaction of **3**‐OTf with a large excess of benzaldehyde (ca. 50 equiv.) was investigated in C_6_D_6_ in the absence of HBpin. This reaction can be shown by in situ NMR monitoring to generate benzyl benzoate, PhC(O)OCH_2_Ph, as the major product. This reaction represents a Nacnac‐supported variant of the well‐known Tischenko reaction,^[^ ^40^ ^]^ through which aldehydes are catalytically converted into esters by an aluminium alkoxide. Indeed both **4**‐OTf and **4**‐Et are found to catalyse the conversion of benzaldehyde to benzyl benzoate at 2 mol% loading, albeit with somewhat different (but unimpressive) turnover frequencies (2.1 and 0.1 h^−1^, respectively; Figures S12 and S13). Given a mechanism for the Tischenko reaction which proceeds in a manner analogous to the classical variant (Scheme 4), the initial reaction of **4**‐OTf with PhCHO would be expected to generate (Nacnac)^Dipp^Al(OTf)(OCH(Ph)OCH_2_Ph) (**5**‐OTf). We therefore attempted to generate this species independently, in order to test its potential reactivity towards HBpin. However, the reaction of **4**‐OTf with one equivalent of benzaldehyde simply generates benzyl benzoate (consuming 50% of the **4**‐OTf), and the attempted hydroalumination of PhC(O)OCH_2_Ph by **3**‐OTf (in a ca. 1:1 ratio) yields only **4**‐OTf (with half of the ester left unreacted). These data speak to the lability of **5**‐OTf under such conditions. Nonetheless, undertaking these reactions in the presence of HBpin yields no trace of PhCH_2_OBpin, implying that in any case **5**‐OTf is not relevant to the chemistry reported by Yang et al.^[^ ^26^ ^]^

Given the lack of competence of **4**‐OTf as a catalytic intermediate in the conversion of PhCHO to PhCH_2_OBpin, we considered alternative pathways which might bring about this hydroboration chemistry. Initially we intended to re‐examine the catalytic reaction of benzaldehyde with HBpin under conditions analogous to those reported by Yang et al. However, in our hands, the background (uncatalysed) reaction occurring between commercially sourced HBpin and benzaldehyde is significant,^[^ ^41^, ^42^ ^]^ and strongly dependent on the purity of both compounds. We were aware that commercially sourced HBpin can contain BH_3_‐derived impurities that can catalyse hydroboration chemistry;^[^ ^43^, ^44^, ^45^ ^]^ our as‐received HBpin (Aldrich) features a quartet signal in the ^11^B NMR spectrum at *δ*_B_ = −13.6 pm consistent with such a species.^[^ ^46^ ^]^ As such, we employed a purification protocol using PPh_3_, to generate Ph_3_P·BH_3_, from which HBpin can then be separated by distillation (distillation alone did not completely remove the impurity).^[^ ^47^, ^48^ ^]^ While this protocol does indeed remove the BH_3_‐containing impurity (Figures S8 and S9), the purified HBpin and freshly distilled PhCHO when combined at the (relatively high) concentrations used in the original study (2 mmol of each in 1 mL of C_6_D_6_) react not in the ‘trace amounts’ reported,^[^ ^26^ ^]^ but in ca. 30% conversion over 1 h (Figures S14 and S15).^[^ ^41^, ^42^ ^]^ As such–both with respect to the current study and more broadly–the reaction of HBpin with benzaldehyde is clearly not a good probe of catalytic activity. With this in mind, we looked at other substrates investigated by Yang et al.^[^ ^26^ ^]^

**Figure 2:**
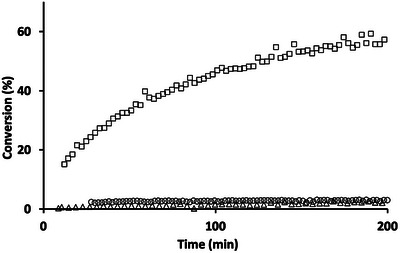
Hydroboration of acetophenone: temporal plots showing the formation of PhC(Me)(H)OBpin: (□) using as‐received HBpin and no **3**‐OTf; (⭕) using purified HBpin and no **3**‐OTf catalyst; (▵) using purified HBpin and 2 mol% **3**‐OTf, as per Yang et al.^[^ ^26^ ^].^

The hydroboration of ketones is generally less facile than aldehydes, and the reaction of acetophenone, PhCOMe, was also investigated by Yang et al. using **3**‐OTf as catalyst (51% reported conversion in benzene, over 6 h at room temperature and 2 mol% catalyst loading).^[^ ^26^ ^]^ We first examined the control reaction, finding very similar conversion (ca. 60% conversion in 200 min) at identical concentrations of reagents to those used by Yang et al., by using commercial HBpin *but no aluminium catalyst* (Figures 2 and S8). On the other hand, using HBpin which had been pre‐cleaned with PPh_3_, we saw essentially no conversion over the same timeframe (Figures 2 and S16).^[^ ^41^, ^42^ ^]^ In general terms, this seems to us to signpost acetophenone as a better test substrate for hydroboration catalysis, since little background conversion is observed. Using this purified HBpin we then examined the reaction with acetophenone catalysed by **3**‐OTf (2 mol% catalyst loading; concentrations of HBpin and PhCOMe as per Yang et al.). Surprisingly, we found very low detectable conversion to PhC(Me)(H)OBpin over 200 min (<< 5%; Figures 2 and S18), in stark contrast to the behaviour previously reported.^[^ ^26^ ^]^

This finding caused us to hypothesise that the true nature of the catalyst in the work reported by Yang et al. is actually a BH_3_ adduct, as has been elegantly revealed for a number of systems by Thomas and coworkers.^[^ ^43^, ^44^ ^]^ Commercially sourced HBpin can contain variable amounts of BH_3_ adduct impurities, and the protocol for purifying the HBpin used in the study by Yang et al. (if any) is not described in the paper or supporting information. In addition, bearing in mind the report by Bakewell et al., that (Nacnac)^Dipp^AlH_2_ (i.e., **3**‐H) degrades HBpin to generate (Nacnac)^Dipp^Al(pin),^[^ ^49^ ^]^ we examined the interaction of HBpin with **3**‐OTf, **4**‐OTf and related complexes. We wished to explore whether catalysis by a BH_3_ adduct could result not only from the use of impure HBpin, but also from the degradation of HBpin caused by the action of aluminium species likely to be present in the reaction mixture.^[^ ^43^, ^44^, ^49^, ^50^, ^51^, ^52^ ^]^

While we have previously demonstrated that **3**‐OTf reacts (reversibly) with {H(9‐BBN)}_2_ to give the corresponding dialkylborohydride complex (Nacnac)^Dipp^Al(OTf){H_2_(9‐BBN)},^[^ ^53^ ^]^ no convincing evidence (either experimental or computational) could be obtained for the formation of a similar borohydride type species between **3**‐OTf and HBpin–consistent with the weaker Lewis acidity of the latter. However, prolonged exposure of clean HBpin to **3**‐H (as reported by Bakewell),^[^ ^49^ ^]^ or to **3**‐OTf or **4**‐OTf, leads to the formation of BH_3_‐containing species, as judged by structural and spectroscopic studies. Thus, in our hands, (Nacnac)^Dipp^AlH_2_ (**3**‐H) reacts with HBpin under pseudo‐catalytic conditions (50–100 equiv. of borane, C_6_D_6_, room temperature) to generate over ca. 30 min the previously reported species (Nacnac)^Dipp^Al(pin),^[^ ^49^ ^]^ together with a BH_3_ adduct which gives rise to a signal (at *δ*_B_ = −13.6 ppm) identical to that found as an impurity in commercial HBpin (Figure S20). Subsequent addition of PPh_3_ to the reaction mixture generates the diagnostic doublet ^11^B{^1^H} NMR signal of Ph_3_P·BH_3_ (at *δ*_B_ = −38.1 ppm; ^1^ *J*_B–P_ = 50 Hz)).^[^ ^47^, ^48^ ^]^ Also generated at extended reaction times (12 h) is (Nacnac)^Dipp^Al(BH_4_)_2_ (*δ*_B_ = −37.0 ppm), a compound which has previously been reported by Harder and coworkers,^[^ ^54^ ^]^ and which we synthesised independently from (Nacnac)^Dipp^AlH_2_ and Me_2_S·BH_3_, and structurally authenticated by X‐ray crystallography (Figure S28). This species presumably arises from partial sequestration of the generated BH_3_ species by **3**‐H.

**3**‐OTf also reacts with HBpin under similar conditions to generate the same BH_3_ adduct (at *δ*_B_ = −13.6 ppm) and a borohydride species (*δ*_B_ = −43.5 ppm) which we postulate to be (Nacnac)^Dipp^Al(OTf)(BH_4_) on the basis of the similarity in the ^11^B chemical shift with those of other species of the type (Nacnac)^Dipp^Al(X)(BH_4_) (Figures S7, S20, and S21).^[^ ^53^ ^]^ A species with an identical ^11^B NMR resonance is generated in the independent reaction of **3**‐OTf with BH_3_·SMe_2_, although in this case isolation of this compound as a pure species proved difficult. Both signals are evident (albeit weakly so) after 5 min reaction time, and grow in continuously over a period of 16 h. **4**‐OTf also generates the same BH_3_ adduct from HBpin under pseudo‐catalytic conditions (Figure S22), with the diagnostic ^11^B NMR resonance at *δ*_B_ = −13.6 ppm being evident after 10 min, and increasing in intensity continuously over a period of 5 days. Interestingly, in this case a second ^11^B NMR signal grows in concurrently at a chemical shift diagnostic of B_2_pin_3_ (*δ*_B_ = 22.0 ppm), and we postulate that **4**‐OTf promotes a redistribution reaction which converts HBpin into BH_3_ and B_2_pin_3_ in a manner well known for HBcat following the work of Baker, Marder, and coworkers.^[^ ^55^ ^]^

Finally, the fact that these HBpin degradation pathways can lead to the generation of catalytically active species can be demonstrated explicitly. Mixing acetophenone, clean HBpin and **3**‐OTf in a 1:1:0.02 ratio generates no PhC(H)MeOBpin over a period of 4 h (as determined by in situ ^1^H NMR monitoring), but leads to 13% conversion (6.5 turnovers) after 12 h. This timeframe for conversion is consistent with the induction period associated with the degradation of HBpin to BH_3_‐containing species by **3**‐OTf.

## Conclusions

The hydroboration of aldehydes and ketones by pinacolborane catalysed by (Nacnac)^Dipp^Al(OTf)H, first reported in 2015,^[^ ^26^ ^]^ represents a highly‐cited landmark in the development of main group compounds for homogeneous catalysis. We show here, however, i) that the mechanism originally proposed for this process involving turnover via Al─O/B─H metathesis at the key intermediate (Nacnac)^Dipp^Al(OTf)(OCH_2_Ph) does not occur– and indeed is thermodynamically viable *only in the reverse direction*; ii) using carefully pre‐purified HBpin, the reaction with acetophenone ‘catalysed’ by (Nacnac)^Dipp^Al(OTf)H actually shows no conversion to PhC(Me)(H)OBpin over the timeframe originally reported (cf. 51% apparent conversion); and iii) that the active species in catalysis is a BH_3_ adduct which arises from the use of impure HBpin, or from the degradation of HBpin caused by the action of aluminium species present in the reaction mixture. More broadly we believe that this study i) calls into question the nature of the true catalyst species in reports of carbonyl hydroboration by aluminium complexes in general; and ii) presents further evidence that the reaction of HBpin with benzaldehyde is not a good probe of catalytic activity, given the significant rate of the background reaction.

## Experimental

Synthetic and characterising data for key compounds, together with representative spectra, temporal plots, and details of crystallographic studies are included in the Supporting Information.^[^ ^56^ ^]^

## Supporting Information

The authors have cited an additional reference within the Supporting Information.^[^ ^57^ ^]^

## Conflict of Interests

The authors declare no conflict of interest.

## Supporting information



Supporting Information

Supporting Information

## Data Availability

The data that support the findings of this study are available in the supplementary material of this article.
