# Supplementary material for: On the Viability of Carbonyl Hydroboration Catalysed by Aluminium Hydrides
Source: Angew Chem Int Ed Engl. 2025 Sep 20;64(46):e202517007. doi: 10.1002/anie.202517007 (PMC12603974; doi:10.1002/anie.202517007)
Supplement: Supplementary file 1 — Supporting Information [file ANIE-64-e202517007-s002.pdf]

On the Viability of Carbonyl Hydroboration Catalysed by Aluminium Hydrides

Alexa Caise, Barnabé Berger, Aidan Murray, Eugene Kolychev, Jamie Hicks, M. Ángeles Fuentes, Job J. C. Struijs, Jose M. Goicoechea and Simon Aldridge

Supporting Information (38 pages)

1. General considerations S2
2. Synthetic procedures and characterizing data for novel compounds S3
3. Representative spectra S11
4. NMR-monitored reactions S18
5. X-ray crystallography S31
6. DFT calculations on the inter-conversion of **3**-OTf and **4**-OTf S37
7. References for supporting information S38

1. General considerations

All manipulations were carried out using standard Schlenk line or dry-box techniques under an atmosphere of dry argon or dinitrogen. Solvents were degassed by sparging with argon and dried by passing through a column of the appropriate drying agent using a commercially available Braun SPS and stored over potassium. NMR spectra were measured in benzene-d_6 or toluene-d_8 which were dried over CaH_2, with the solvent then being distilled under reduced pressure, degassed by three freeze-pump-thaw cycles and stored under argon in Teflon valve ampoules. NMR samples were prepared under argon in 5 mm Wilmad 507-PP tubes fitted with J. Young Teflon valves. NMR spectra were measured on a Bruker Avance III HD Nanobay 400 MHz NMR spectrometer equipped with a 9.4 T magnet, Bruker Avance III 500 MHz NMR spectrometer equipped with a 11.75 T magnet or a Bruker Avance III NMR 500 MHz NMR spectrometer equipped with a 11.75 T magnet and a ^{13}C detect cryoprobe. ^{1}H and ^{13}C NMR spectra were referenced internally to residual protio-solvent (^{1}H) or solvent (^{13}C) resonances and are reported relative to tetramethylsilane (δ = 0 ppm). ^{11}B and ^{19}F NMR spectra were referenced with respect to $BF_3{\cdot}OEt_2$ and CF_3COOH, respectively. Chemical shifts are quoted in δ (ppm) and coupling constants in Hz. Infra-red spectra were measured on a Nicolet 500 FT-IR spectrometer. Samples were measured as a Nujol mull and were prepared inside a glovebox before being sealed in an airtight cell. Elemental analyses were carried out at London Metropolitan University.

The syntheses of **3**-OTf,[S1] **3**-Cl,[S2] **3**-Et,[S2] **3**-Me[S2] and (Nacnac)$^{Dipp}GaH_2$[S3] were carried out by the literature routes. The syntheses of (Nacnac)^{Dipp}AlH(OCH_2Ph) (**4**-H) and (Nacnac)^{Dipp}Al(OCH_2Ph)$_2$ (**4**-OCH_2Ph) by the insertion of one or two equivalents of benzaldehyde into the corresponding aluminium dihydride (**3**-H) have previously been reported.[S4] The synthesis of of (Nacnac)^{Dipp}Al(BH_4)$_2$ has previously been reported via a different route to that described below.[S5]

2. Synthetic procedures and characterizing data for novel compounds

(Nacnac)^{Dipp}Al(OTf)(OCH$_2$Ph) (4-OTf): To a solution of **3**-OTf (0.50 g, 0.84 mmol) in toluene (5 mL) at room temperature was added dropwise benzaldehyde (98 µL, 0.967 mmol) with stirring. The reaction mixture was stirred for 12 h, and volatiles then removed *in vacuo* from the resulting yellow solution to yield a yellow oil. The oil was extracted into *n*-hexane, and volatiles were again removed *in vacuo* to yield a yellow powder. The powder was extracted into *n*-hexane, filtered and concentrated to the point of incipient crystallisation. Storage of this solution at −30°C for several days yielded large yellow crystals of **4**-OTf, which were isolated and dried *in vacuo*. Yield: 0.36 g, 61%. Crystals suitable for X-ray crystallography could be obtained from the storage of a concentrated solution in pentane at −30°C for several days. **^{1}H NMR** (400 MHz, benzene-d$_6$, 298 K): δ_H 1.02 (6H, d, $^3J_{HH}$ = 6.8 Hz, CH$_3$ of Dipp ^{i}Pr), 1.16 (12H, apparent t, $^3J_{HH}$ = 6.8 Hz, 2 overlapping CH$_3$ of Dipp ^{i}Pr), 1.47 (6H, d, $^3J_{HH}$ = 6.8 Hz, CH$_3$ of Dipp ^{i}Pr), 1.54 (6H, s, CH$_3$ of β-diketiminato backbone), 3.27 (2H, sept, $^3J_{HH}$ = 6.8 Hz, CH of Dipp ^{i}Pr), 3.29 (2H, sept, $^3J_{HH}$ = 6.8 Hz, CH of Dipp ^{i}Pr), 5.04 (1H, s, γ-CH), 5.09 (2H, s, CH$_2$ of OCH$_2$Ph), 7.05 – 7.23 (11H, m, aromatic CH of Dipp and OCH$_2$Ph). **^{13}C{^{1}H} NMR** (101 MHz, benzene-d$_6$, 298 K): δ_C 23.5 (CH$_3$ of β-diketiminato backbone), 24.5, 24.6, 24.8, 24.9 (CH$_3$ of Dipp ^{i}Pr), 28.5, 28.7 (CH of Dipp ^{i}Pr), 65.3 (CH$_2$ of OCH$_2$Ph), 99.1 (γ-CH), 124.9 (*m*-CH of Dipp, 2 overlapping signals), 125.1 (*o*-ArC of OCH$_2$Ph), 126.4 (*p*-ArC of OCH$_2$Ph), 127.4 (*p*-ArC of Dipp), 128.3 (*m*-ArC of OCH$_2$Ph), 138.3 (*ipso*-ArC of Dipp), 144.3, 144.7 (*o*-ArC of Dipp), 144.2 (*ipso*-ArC of OCH$_2$Ph), 173.5 (NC). **^{19}F NMR** (376 MHz, benzene-d$_6$, 298 K): δ_F −77.4. **^{27}Al NMR** (104 MHz, benzene-d$_6$, 298 K): δ_{Al} not observed. **EI-MS**: *m/z* calc for $C_{37}H_{48}N_2AlF_3O_4S$ ([M]$^+$) 700.3097, meas. 700.3075 (5%), calc for ([M−OCH$_2$Ph]$^+$) 593.2605, meas. 593.2557 (2%). **Elemental microanalysis**: calc. for $C_{37}H_{48}N_2AlF_3O_4S$: C 63.41% H 6.90% N 4.00% meas. C 63.36% H 6.63% N 4.21%.

(Nacnac)^{Dipp}Al(Et)(OCH_2Ph) (4-Et): To a solution of **3**-Et (0.30 g, 0.63 mmol) in toluene (10 mL) at room temperature was added dropwise benzyl alcohol (98 µL, 0.95 mmol), leading to the evolution of hydrogen. The reaction mixture was stirred for 3 h, over which time evolution of gas ceased; volatiles were then removed *in vacuo* to yield a colourless oil. The oil was extracted into *n*-hexane, concentrated until the volume reached *ca.* 0.5 mL, and cooled to −30°C. Storage of this solution at this temperature yielded large single crystals of **4**-Et, suitable for X-ray crystallography. Yield: 0.31 g, 85%. **^{1}H NMR** (400 MHz, benzene-d_6, 298 K): δ_H −0.08 (2H, q, $^3J_{HH}$ = 8.2 Hz, $CH_3C\underline{H}_2Al$), 0.96 (3H, t, $^3J_{HH}$ = 8.2 Hz, $C\underline{H}_3CH_2Al$), 1.09, 1.10, 1.19, 1.31 (6H, d, $^3J_{HH}$ = 6.8 Hz, CH_3 of Dipp ^{i}Pr), 1.60 (6H, s, CH_3 of β-diketiminato backbone), 3.20 (2H, sept, $^3J_{HH}$ = 6.8 Hz, CH of Dipp ^{i}Pr), 3.54 (2H, sept, $^3J_{HH}$ = 6.8 Hz, CH of Dipp ^{i}Pr), 5.02 (1H, s, γ-CH), 5.14 (2H, s, CH_2 of OCH_2Ph), 7.07 – 7.16 (7H, m, aromatic CH of Dipp and *p*-CH of OBn), 7.27 (2H, apparent t, $^3J_{HH}$ = 7.5 Hz, *m*-CH of OCH_2Ph), 7.58 (2H, dm, $^3J_{HH}$ = 7.8 Hz, *o*-CH of OBn). **^{13}C{^{1}H} NMR** (101 MHz, benzene-d_6, 298 K): δ_C −3.4 (br, $CH_3\underline{C}H_2Al$), 9.4 ($\underline{C}H_3CH_2Al$), 23.3 (CH_3 of β-diketiminato backbone), 24.4, 24.6, 24.8, 25.6 (CH_3 of Dipp ^{i}Pr), 28.0, 28.9 (CH of Dipp ^{i}Pr), 65.5 (CH_2 of OCH_2Ph), 98.1 (γ-CH), 124.1, 124.9 (*m*-ArC of Dipp), 125.7 (*o*-ArC of OBn), 126.1 (*p*-ArC of OCH_2Ph), 127.4 (*p*-ArC of Dipp), 128.2 (*m*-ArC of OCH_2Ph), 141.0 (*ipso*-ArC of Dipp), 143.4, 145.4 (*o*-ArC of Dipp), 146.3 (*ipso*-ArC of OCH_2Ph), 170.0 (NC). **^{27}Al NMR** (104 MHz, benzene-d_6, 298 K): δ_{Al} 111.4. **EI-MS**: *m/z* calc for $C_{36}H_{48}N_2AlO$ ($[M-CH_2CH_3]^+$) 551.3582, meas. 551.3589 (50%).

(Nacnac)^{Dipp}Al(Cl)(OCH$_2$Ph) (4-Cl): To a solution of **3**-Cl (0.375 g, 0.78 mmol) in toluene (10 mL) at room temperature was added dropwise benzaldehyde (88 µL, 0.86 mmol). The resulting solution was stirred for 12 h at room temperature and volatiles then removed *in vacuo*, yielding an orange oil. This oil was extracted into minimal toluene, filtered and concentrated to the point of incipient crystallisation. Storage of this solution at −30°C for one week yielded colourless crystals of **4**-Cl suitable for X-ray diffraction. Yield: 0.35 g, 77%. **^{1}H NMR** (400 MHz, benzene-d$_6$, 298 K): δ_H 1.06, 1.17, 1.29, 1.44 (6H, d, $^3J_{HH}$ = 6.8 Hz, CH_3 of Dipp ^{i}Pr), 1.54 (6H, s, CH_3 of β-diketiminato backbone), 3.38 (2H, sept, $^3J_{HH}$ = 6.8 Hz, CH of Dipp ^{i}Pr), 3.55 (2H, sept, $^3J_{HH}$ = 6.8 Hz, CH of Dipp ^{i}Pr), 4.64 (2H, s, CH_2 of OCH_2Ph), 4.89 (1H, s, γ-CH), 6.71 (2H, m, aromatic CH of OCH_2Ph), 6.97 – 7.22 (9H, m, aromatic CH of Dipp and OBn). **^{13}C{^{1}H} NMR** (101 MHz, benzene-d$_6$, 298 K): δ_C 23.4 (CH_3 of β-diketiminato backbone), 24.3, 24.9, 25.0, 26.4 (CH_3 of Dipp ^{i}Pr), 28.3, 29.1 (CH of Dipp ^{i}Pr), 65.4 (CH_2 of OBn), 98.0 (γ-CH), 124.5, 125.2 (*m*-ArC of Dipp), 125.9 (*p*-ArC of OBn), 126.1 (*o*-ArC of OBn), 127.9 (*p*-ArC of Dipp), 128.0 (*m*-ArC of OBn), 139.0 (*ipso*-ArC of Dipp), 144.5 (*ipso*-ArC of OBn), 144.6, 145.4 (*o*-ArC of Dipp), 171.6 (NC). **^{27}Al NMR** (104 MHz, benzene-d$_6$, 298 K): δ_{Al} 100.6. **EI-MS**: *m/z* calc for $C_{36}H_{48}AlClN_2O$ ([M]$^+$) 586.3265, meas. 586.3277 (100%).

(Nacnac)^{Dipp}Ga(OTf)H: MeOTf (0.30 g, 2.04 mmol) was added dropwise to a solution of (Nacnac)^{Dipp}GaH$_2$ (1.0 g, 2.04 mmol) in toluene (15 mL) at 0°C. The colourless reaction mixture was allowed to warm to room temperature and stirred for 72 h, followed by removal of volatiles *in vacuo.* The resulting white solid residue was re-extracted into minimal toluene, filtered and stored at −30°C, yielding large colourless single crystals of (Nacnac)^{Dipp}Ga(OTf)H (as the toluene solvate) suitable for X-ray crystallography. Yield: 0.71 g (55%). **^{1}H NMR** (400 MHz, benzene-d$_6$, 298 K): δ_H 1.06, 1.10, 1.28 (6H, br m, CH$_3$ of Dipp ^{i}Pr), 1.50 (6H, s, CH$_3$ of β-diketiminato backbone), 1.52 (6H, br. m, CH$_3$ of Dipp ^{i}Pr), 2.99, 3.25 (2H, br. m, CH of Dipp ^{i}Pr), 4.76 (1H, s, γ-CH), 5.62 (1H, br. s, GaH), 6.96 – 7.15 (6H, br. m, aromatic CH). **^{13}C{^{1}H} NMR** (101 MHz, benzene-d$_6$, 298 K): δ_C 23.1 (CH$_3$ of β-diketiminato backbone), 24.4, 24.9, ((br.) CH$_3$ of Dipp ^{i}Pr), 28.6 (CH of Dipp ^{i}Pr), 96.7 (γ-CH), 124.9, 125.2, 128.5, 137.7, 143.5, 144.4 (ArC of Dipp), 171.2 (NC). Resonance for CF$_3$ not observed. **^{19}F NMR** (376 MHz, benzene-d$_6$, 298 K): δ_F −77.5. **^{1}H NMR** (400 MHz, tetrahydrofuran-d$_8$, 298 K): δ_H 1.21, 1.29 (12H, d, $^3J_{HH}$ = 6.8 Hz, CH$_3$ of Dipp ^{i}Pr), 1.92 (6H, s, CH$_3$ of β-diketiminato backbone), 3.11 (4H, sept, $^3J_{HH}$ = 6.8 Hz, CH of Dipp ^{i}Pr), 5.29 (1H, br s, GaH), 5.36 (1H, s, γ-CH), 7.23 – 7.30 (6H, m, aromatic CH). **^{13}C{^{1}H} NMR** (126 MHz, tetrahydrofuran-d$_8$, 298 K): δ_C 23.6 (CH$_3$ of β-diketiminato backbone), 24.8, 25.2 (CH$_3$ of Dipp ^{i}Pr), 29.2 (CH of Dipp ^{i}Pr), 97.6 (γ-CH), 125.1 (ArC of Dipp), 128.5 (ArC of Dipp), 137.7 (ArC of Dipp), 143.6 (br., ArC of Dipp), 144.4 (br., ArC of Dipp), 172.2 (NC). **^{19}F NMR** (376 MHz, tetrahydrofuran-d$_8$, 298 K): δ_F –78.51. **EI-MS**: *m/z* calc for $C_{30}H_{42}N_2GaF_3O_3S$ ([M]$^+$) 636.2119, meas. 636.214 (4%). **IR**: (nujol/cm^{-1}) $\upsilon_{Ga\text{-}H}$ 1994 (s). **Elemental microanalysis**: calc. for $C_{30}H_{42}N_2GaO_3F_3S$: C 56.53% H 6.64% N 4.39% meas. C 56.63% H 6.76% N 4.48%.

(Nacnac)^{Dipp}Ga(H)(OCH$_2$Ph): To a solution of (Nacnac)^{Dipp}GaH$_2$ (0.70 g, 1.43 mmol) in toluene (5 mL) in an ampoule fitted with a J. Young's valve was added benzaldehyde (163 µL, 1.58 mmol). The reaction mixture was heated at 80°C for 3 weeks. Volatiles were then removed *in vacuo* and the resulting oil extracted into *n*-hexane. Filtration, concentration and storage of the resultant solution at −30°C yielded colourless crystals of (Nacnac)^{Dipp}Ga(H)(OCH$_2$Ph) suitable for X-ray diffraction over a period of one week. Yield: 0.26 g, 31%. Monitoring of the reaction mixture by ^{1}H NMR spectroscopy indicated that conversion to the product reaches > 95% after 3 weeks; the low isolated yield reflects the high solubility of (Nacnac)^{Dipp}Ga(H)(OCH$_2$Ph) in compatible alkane solvents. **^{1}H NMR** (400 MHz, benzene-d$_6$, 298 K): δ_H 1.11, 1.18, 1.23, 1.43 (6H, d, $^3J_{HH}$ = 6.7 Hz, CH$_3$ of Dipp ^{i}Pr), 1.58 (6H, s, CH$_3$ of β-diketiminato backbone), 3.25 (2H, sept, $^3J_{HH}$ = 6.7 Hz, CH of Dipp ^{i}Pr), 3.54 (2H, sept, $^3J_{HH}$ = 6.7 Hz, CH of Dipp ^{i}Pr), 4.60 (2H, s, CH$_2$ of OBn), 4.78 (1H, s, γ-CH), 5.28 (1H, br s, GaH), 6.76 (2H, m, aromatic CH of OBn), 6.99 – 7.18 (9H, m, aromatic CH of OBn and Dipp). **^{13}C{^{1}H} NMR** (101 MHz, benzene-d$_6$, 298 K): δ_C 23.1 (CH$_3$ of β-diketiminato backbone), 24.6, 24.7, 24.9, 25.7 (CH$_3$ of Dipp ^{i}Pr), 28.3, 28.8 (CH of Dipp ^{i}Pr), 68.0 (CH$_2$ of OBn), 95.1 (γ-CH), 124.5, 124.7 (*m*-ArC of Dipp), 125.7 (*p*-ArC of OBn), 126.1 (*o*-ArC of OBn), 127.4 (*p*-ArC of Dipp), 128.0 (*m*-ArC of OBn), 140.4 (*ipso*-ArC of Dipp), 143.7, 145.1 (*o*-ArC of Dipp), 146.1 (*ipso*-ArC of OBn), 169.0 (NC). **IR**: (nujol/cm^{-1}) $\nu_{Ga\text{-}H}$ 1933 (s). **EI-MS**: *m/z* calc for C$_{36}$H$_{48}$N$_2$GaO ([M-H]$^+$) 593.3017, meas. 593.2992 (10%).

(Nacnac)^{Dipp}Al(BH$_4$)$_2$:[S5] To a solution of **3**-H (0.10 g, 0.22 mmol) in toluene (2 mL) at room temperature was added neat $BH_3{\cdot}SMe_2$ (100 µL, 1.05 mmol) and the resulting mixture stirred for 12 h. Volatiles were removed *in vacuo* and the crude product redissolved in toluene, filtered, concentrated to incipient crystallization point and stored at -30°C over 2 days to afford colorless crystals of (Nacnac)^{Dipp}Al(BH$_4$)$_2$ suitable for X-ray crystallography. Spectroscopic data matched the reported literature values.[S5] **^{1}H NMR** (400 MHz, benzene-d$_6$, 298 K): δ_H 1.06 (d, 12H), 1.41 (d, 12H), 1.51 (s, 6H), 3.29 (sept, 4H), 4.92 (s, 1H), 7.15 - 7.07 (m, 6H). **^{11}B{^{1}H} NMR** (128 MHz, benzene-d$_6$, 298 K): δ_B -36.6.

Identification of (Nacnac)^{Dipp}Al(OTf)(BH_4): To a solution of **3**-OTf (0.05 g, 0.08 mmol) in toluene (2 mL) at room temperature was added dropwise $BH_3 \cdot SMe_2$ (50 µL, 0.53 mmol) and the reaction mixture stirred for 12 h. Volatiles were removed in vacuo and the crude product redissolved in toluene, filtered, concentrated to incipient crystallization point and stored at -30°C for several days to afford a colourless solid identified as (Nacnac)^{Dipp}Al(OTf)(BH_4). **^{1}H NMR** (400 MHz, benzene-d$_6$, 298 K): δ_H 1.02 (d, 6H), 1.22 (d, 6H), 1.25 (d, 6H), 1.45 (d, 6H), 1.53 (s, 6H), 3.00 (sept, 2H), 3.49 (sept, 2H), 5.08 (s, 1H), 7.15 - 7.01 (m, 6H). **^{11}B{^{1}H} NMR** (128 MHz, benzene-d$_6$, 298 K): δ_B -43.5.

Protocol for the purification of HBpin using PPh_3: To a sample of PPh_3 (8 g, 30.50 mmol) in one arm of a flame dried H-tube was added by syringe commercial HBpin (Aldrich; 20 mL, 137.83 mmol) and the reaction mixture stirred at room temperature with periodic monitoring of aliquots by multi-nuclear NMR spectroscopy. After 1 week, ^{11}B NMR monitoring indicated complete consumption of the BH_3-containing impurity (δ_B = -13.6 ppm) and formation of $Ph_3P{\cdot}BH_3$ (δ_B = -38.1 ppm).[S6] Distillation at room temperature under static vacuum using liquid nitrogen as a cooling agent afforded BH_3-free HBpin in the second arm of the H-tube, which was subsequently stored in a J. Young's ampoule at 7°C. **^1H NMR** (400 MHz, benzene-d_6, 298 K): δ_H 4.21 (pcq, 1H, BH), 1.00 (s, 12H, Me). **$^{11}B\{^1H\}$ NMR** (128 MHz, benzene-d_6, 298 K) δ_B 28.4.

3. Representative spectra

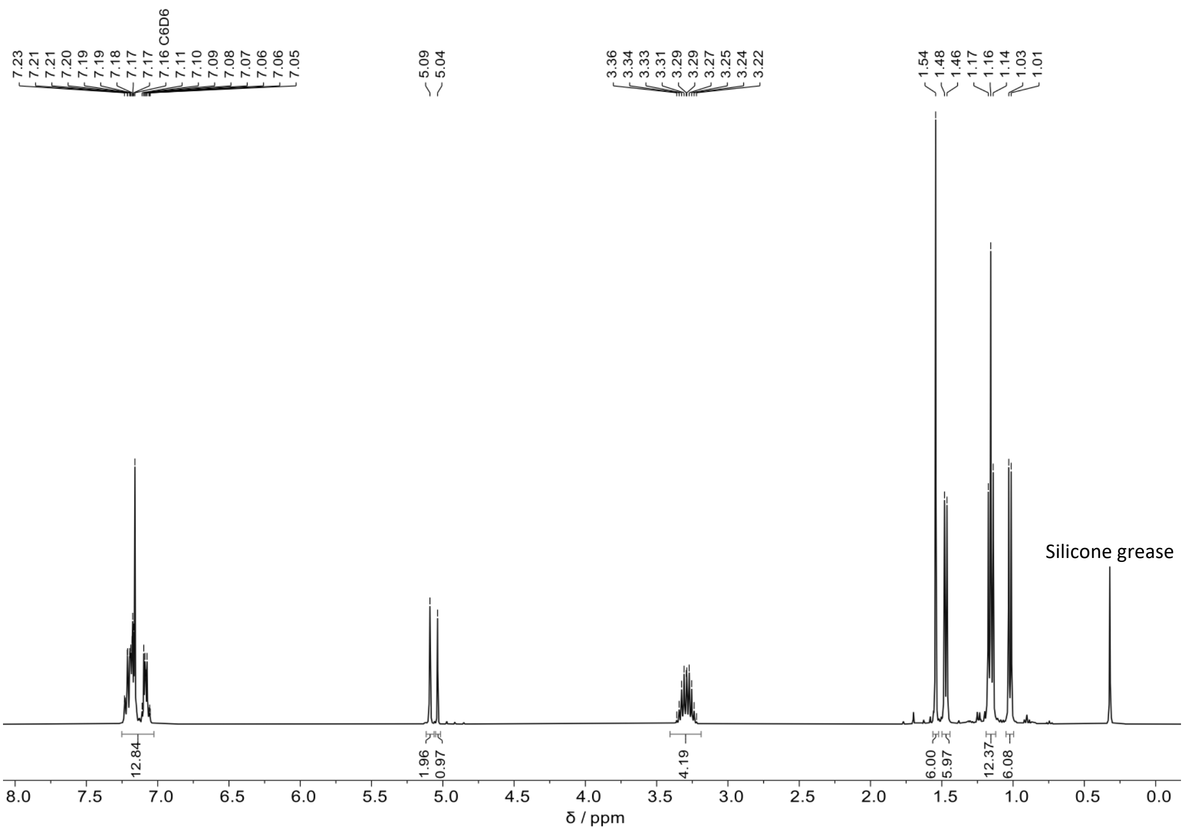

Figure S1: ^{1}H NMR spectrum of (Nacnac)^{Dipp}Al(OTf)(OCH$_2$Ph) (**4**-OTf) in benzene-d$_6$.

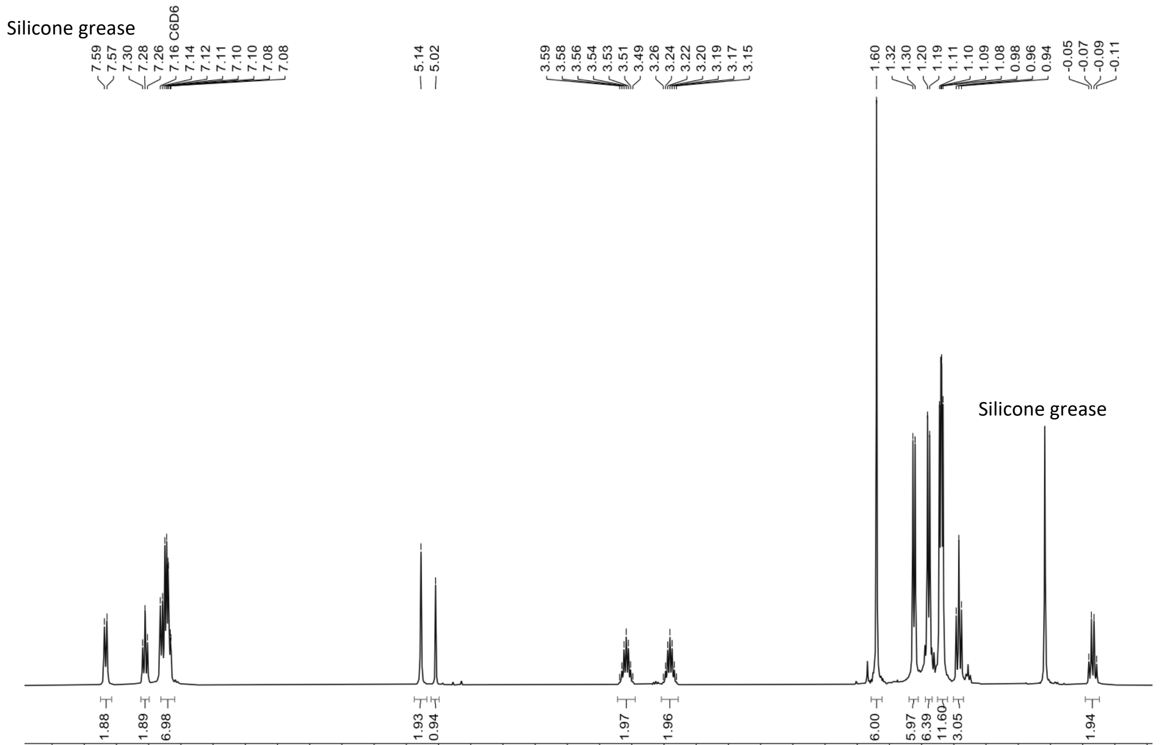

Figure S2: ^{1}H NMR spectrum of (Nacnac)^{Dipp}Al(Et)(OCH$_2$Ph) (**4**-Et) in benzene-d$_6$.

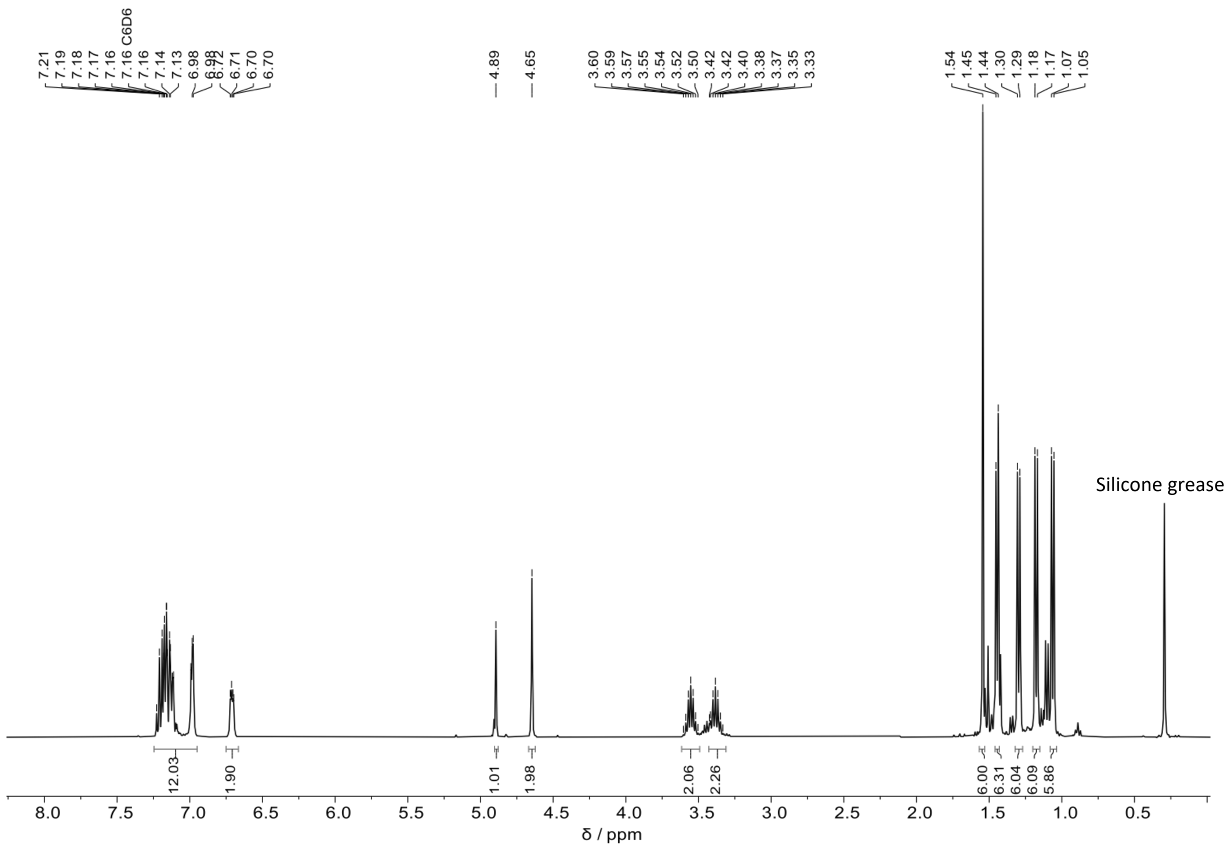

Figure S3: ^{1}H NMR spectrum of (Nacnac)^{Dipp}Al(Cl)(OCH_2Ph) (**4**-Cl) in benzene-d_6.

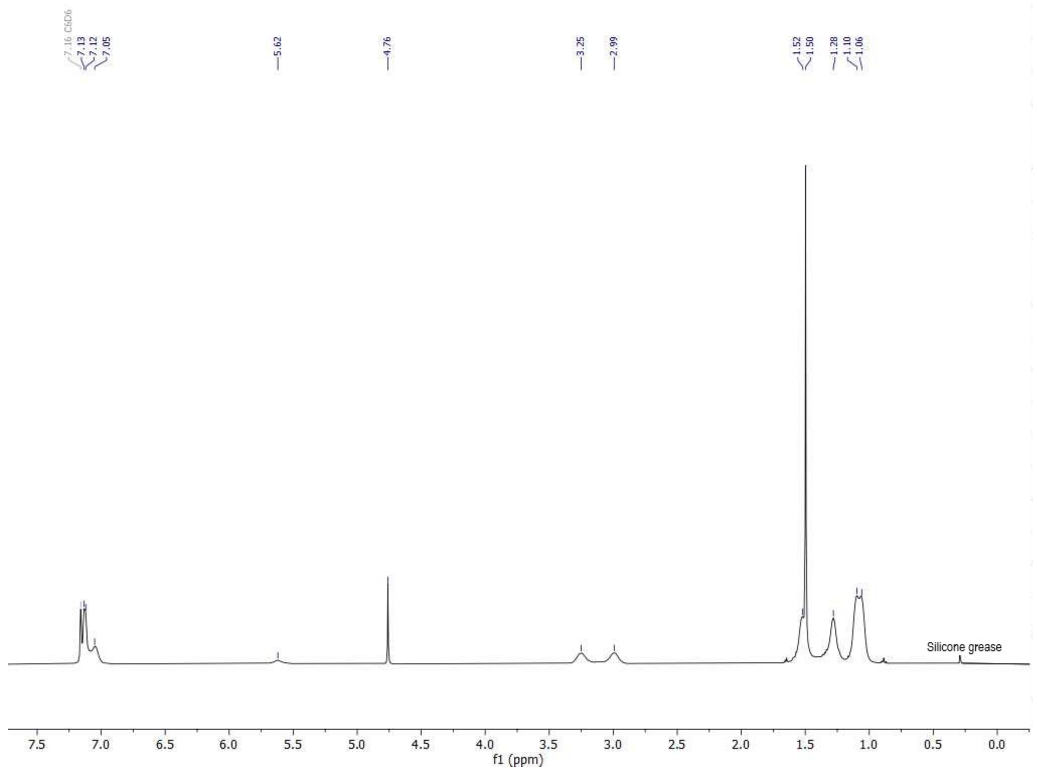

Figure S4: ^{1}H NMR spectrum of (Nacnac)^{Dipp}Ga(OTf)H in benzene-d_6.

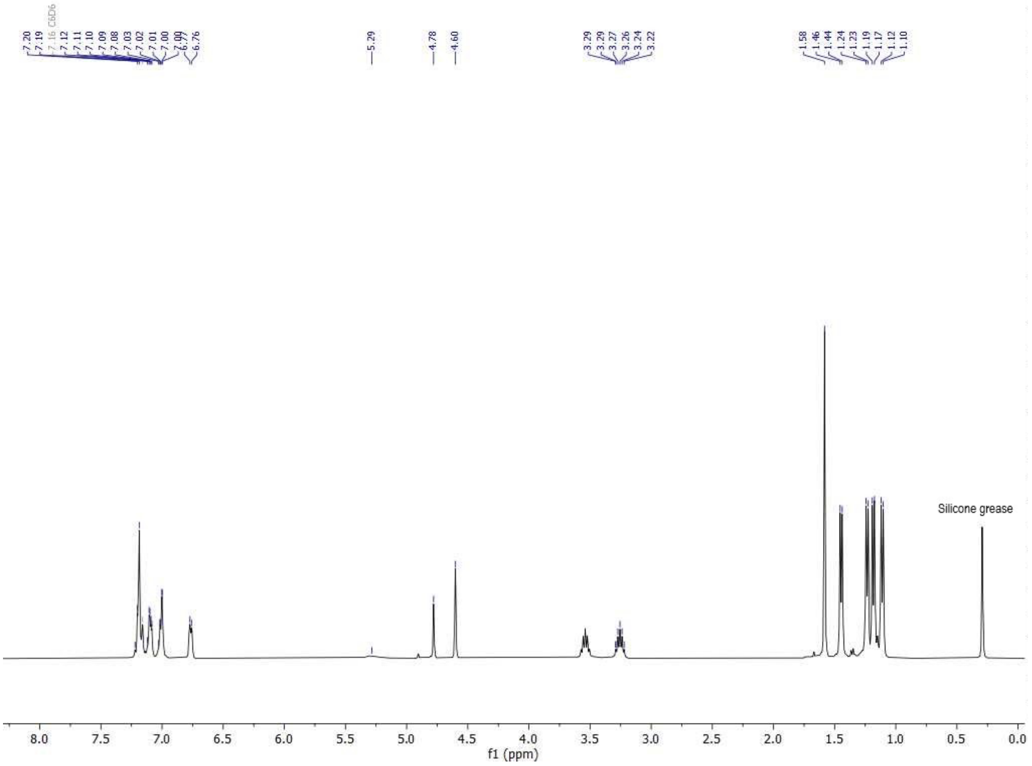

Figure S5: ^{1}H NMR spectrum of (Nacnac)^{Dipp}Ga(H)(OCH_2Ph) in benzene-d$_6$.

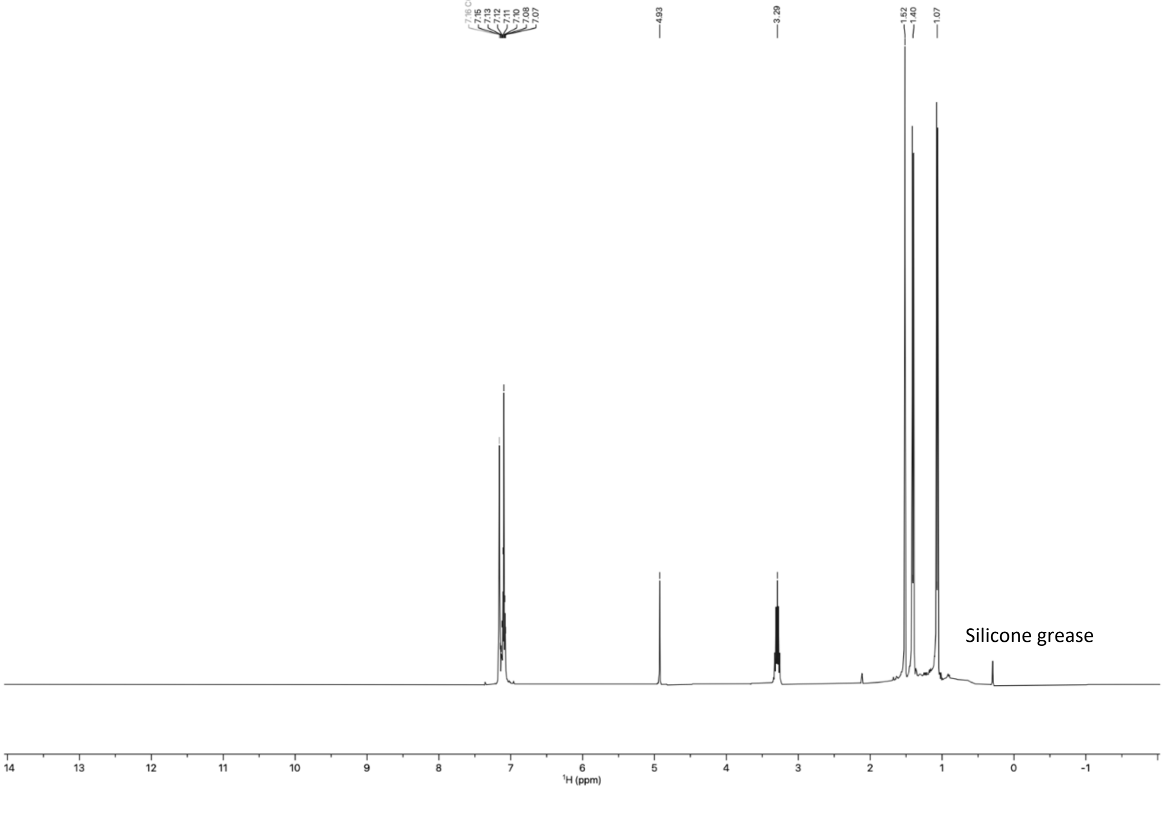

Figure S6a: ^{1}H NMR spectrum of (Nacnac)^{Dipp}Al(BH_4)$_2$ in benzene-d$_6$.

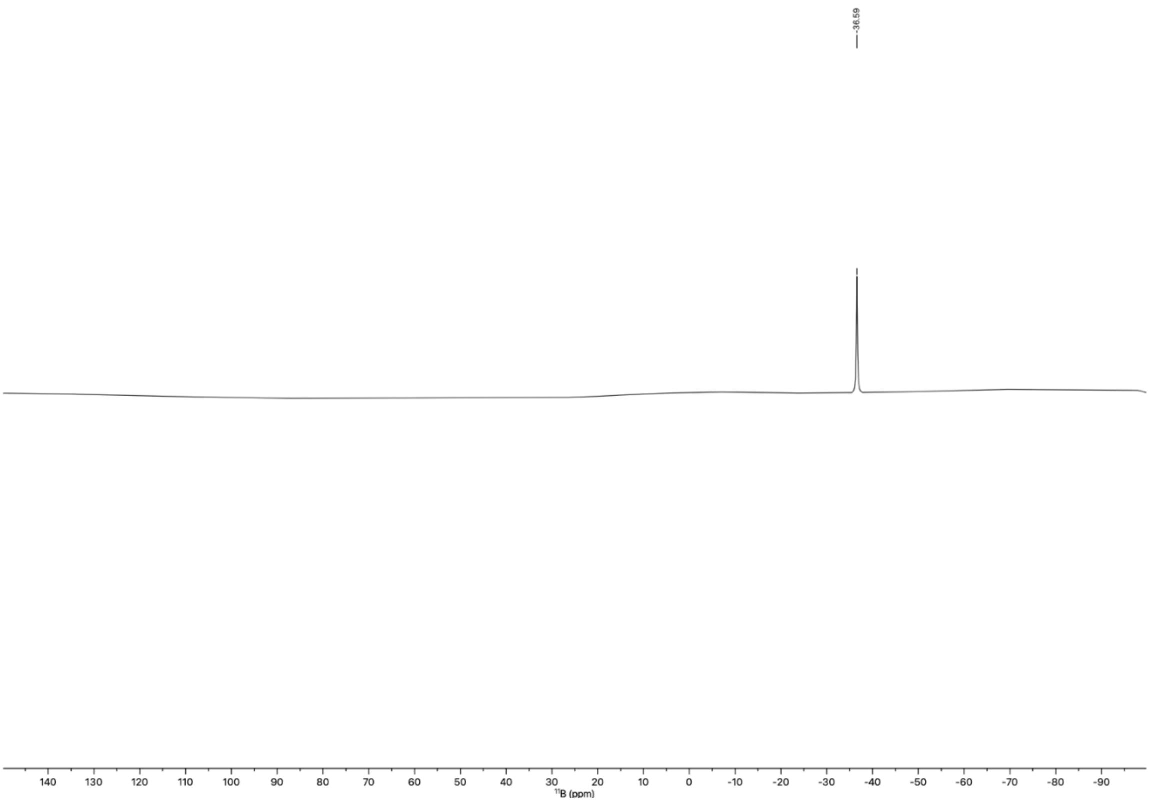

Figure S6b: $^{11}B\{^1H\}$ NMR spectrum of (Nacnac)^{Dipp}Al(BH_4)$_2$ in benzene-d$_6$.

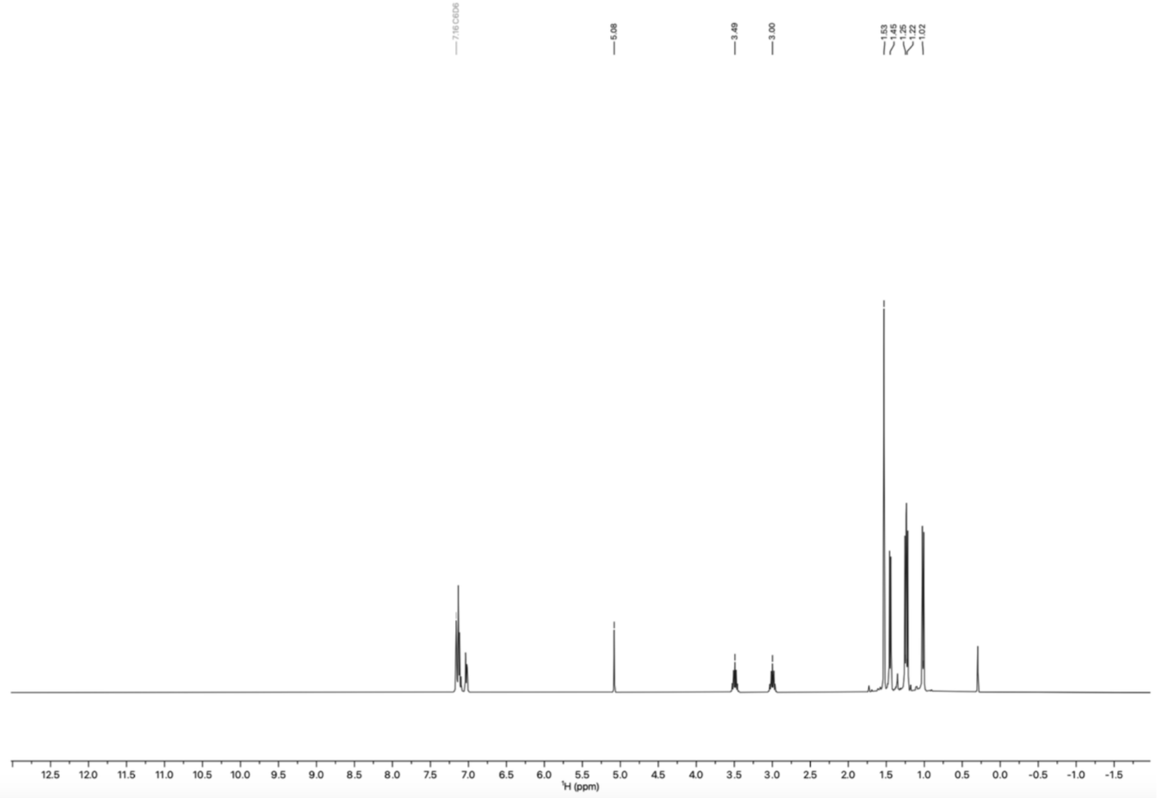

Figure S7a: ^1H NMR spectrum of (Nacnac)^{Dipp}Al(OTf)(BH_4) in benzene-d$_6$.

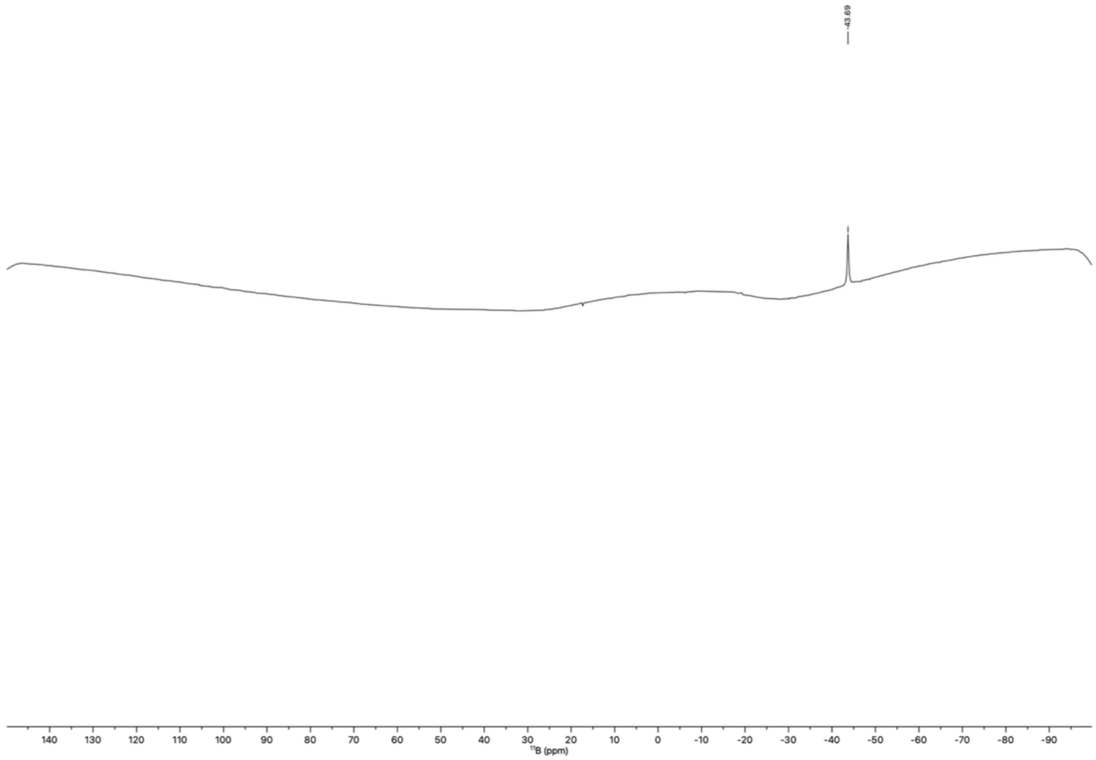

Figure S7b: $^{11}B\{^1H\}$ NMR spectrum of (Nacnac)^{Dipp}Al(OTf)(BH_4) in benzene-d_6.

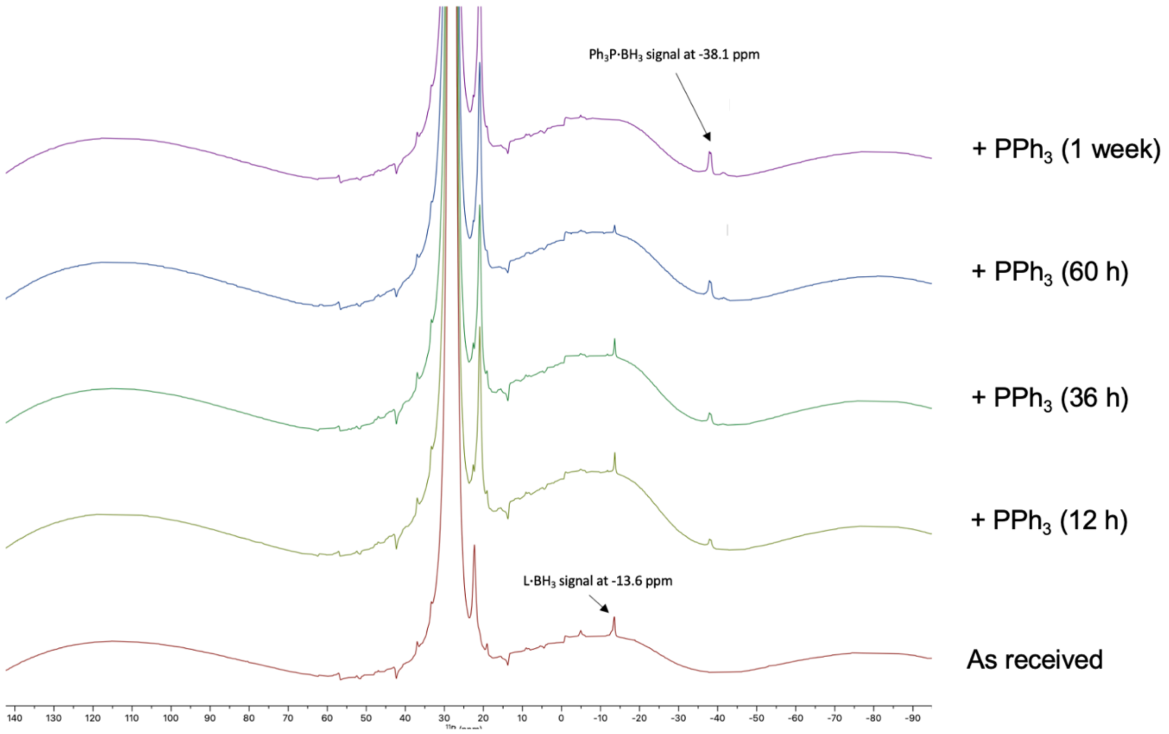

Figure S8: $^{11}B\{^{1}H\}$ NMR spectra of commercial HBpin (Aldrich) as received, and in the presence of PPh_3.

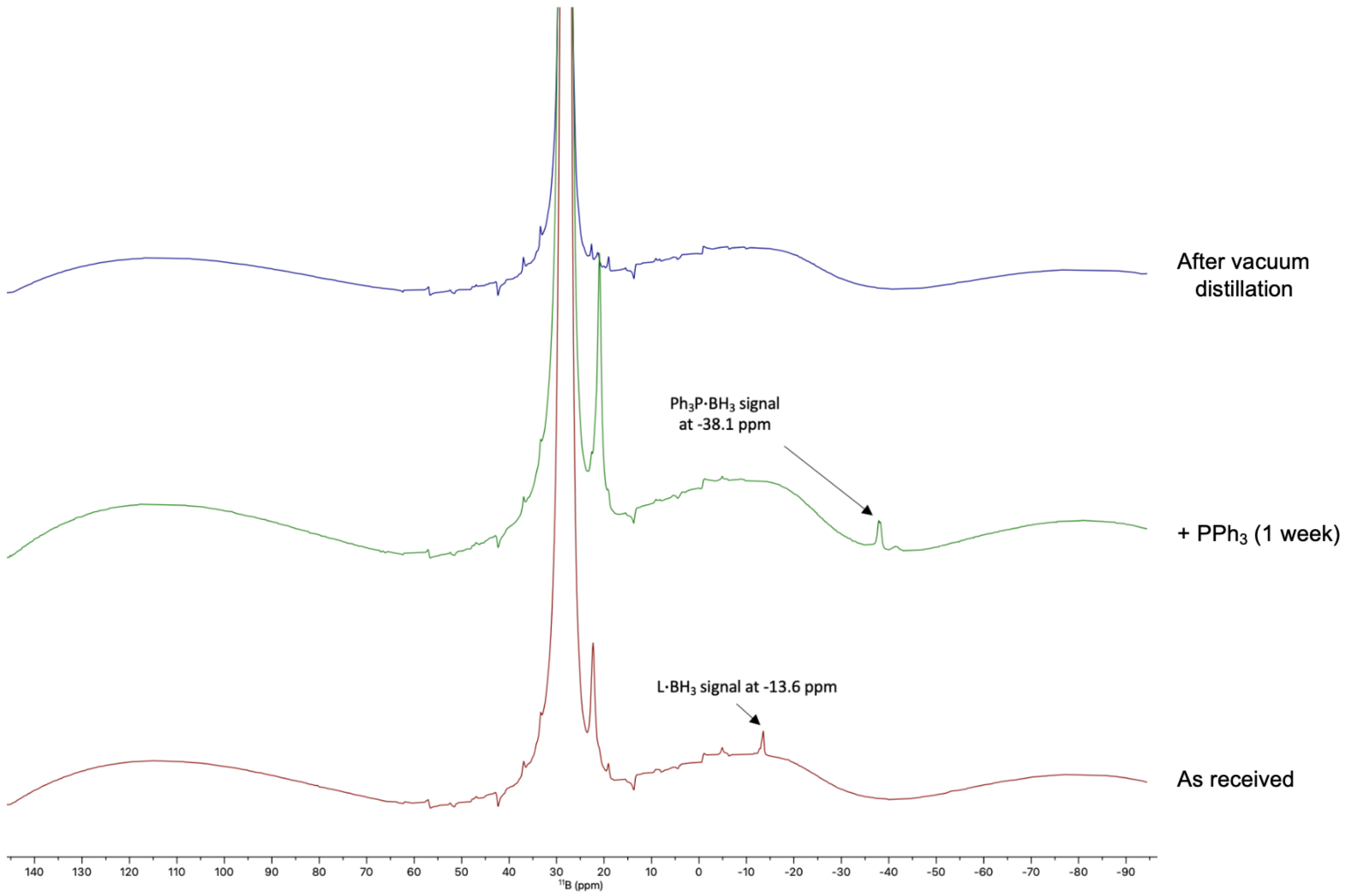

Figure S9: $^{11}B\{^{1}H\}$ NMR spectra showing the purification of commercial HBpin (Aldrich) using PPh_3.

4. NMR-monitored reactions

(i) Assessment of the rate of insertion of benzaldehyde into Al-H bonds: To an NMR tube fitted with a J. Young's valve was added (Nacnac)^{Dipp}Al(X)H (X = Et, Me, H, OCH_2Ph, Cl) (0.063 mmol), benzene-d_6 (0.5 mL) and benzaldehyde (7 µL, 0.069 mmol). The progress of the reaction was monitored by ^{1}H NMR spectroscopy (Figure S10). In the case of (Nacnac)^{Dipp}Al(OTf)H the reaction proceeds very rapidly at room temperature, such that it has gone to completion before an NMR spectrum can be acquired.

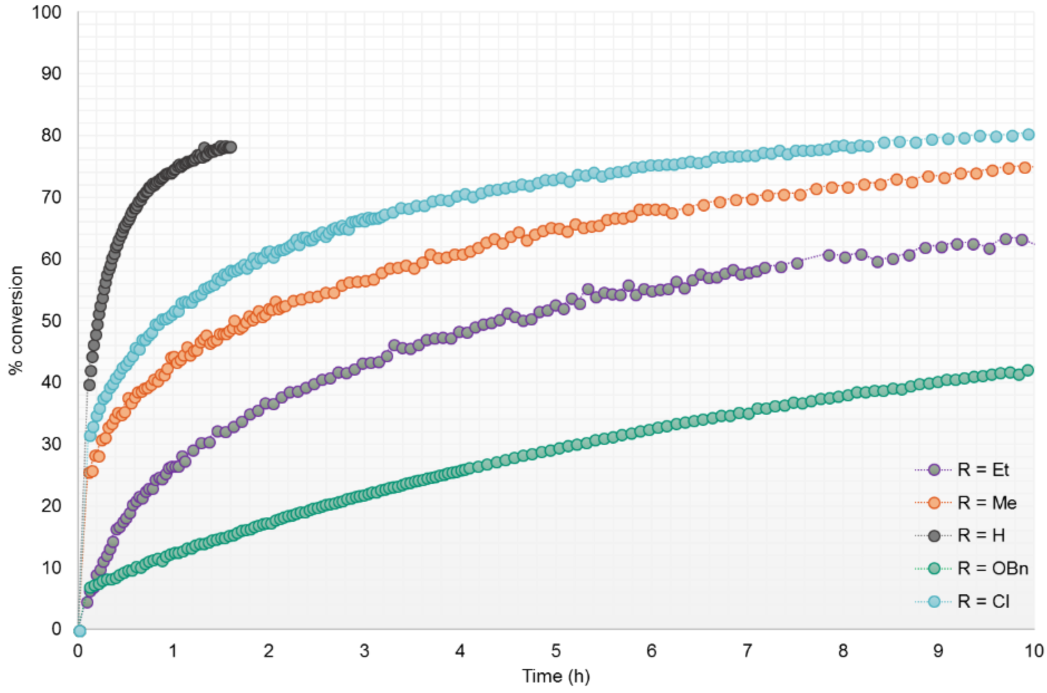

Figure S10: Temporal plot showing the formation of (Nacnac)^{Dipp}Al(X)(OCH_2Ph) vs. time via the insertion of benzaldehyde into the Al-H bond of the corresponding hydrides (Nacnac)^{Dipp}Al(X)H (X = Et, Me, H, OCH_2Ph, Cl) (T = 298 K, t = 0 to 10 h).[S4]

(iii) Assessment of the rate of reaction of the 'reverse' reaction of 3-OTf with $PhCH_2OBpin$ to give 4-OTf: To a solution of $PhCH_2OBpin$ in benzene-d_6 (0.5 mL of a ca. 0.2 M solution, 0.1 mmol) was added **3**-OTf (0.019 mmol), and the progress of the reaction monitored by ^1H NMR spectroscopy.

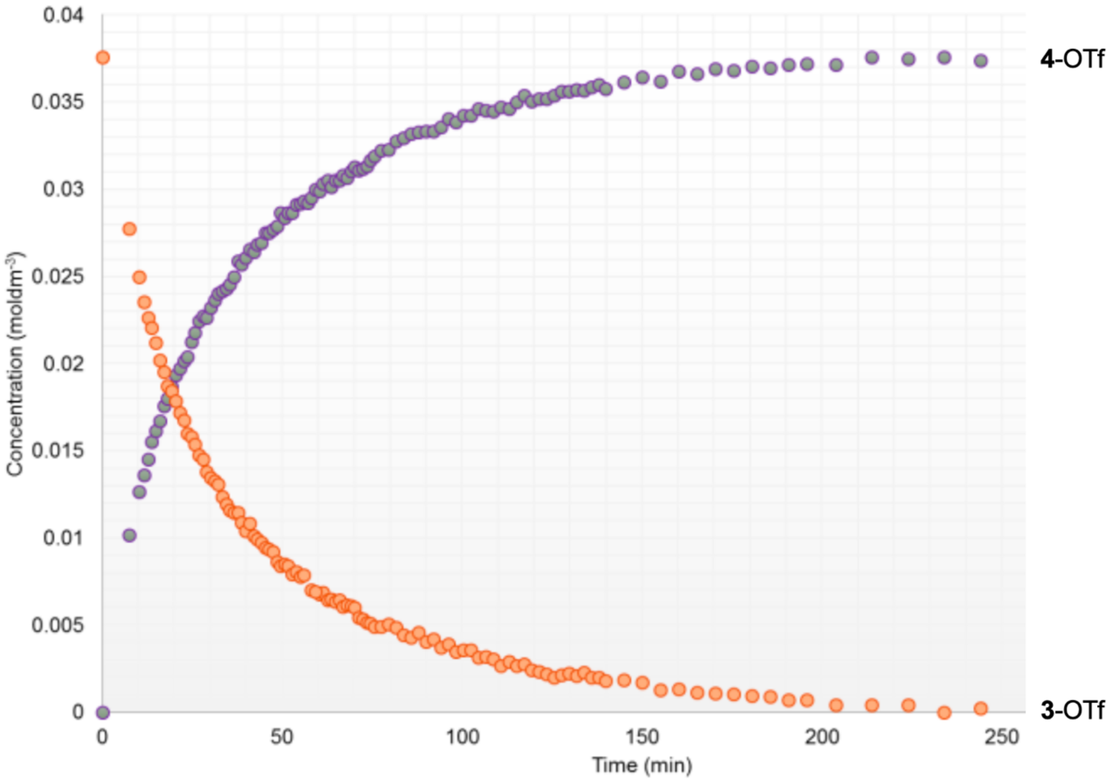

Figure S11: Temporal plot showing the conversion of **3**-OTf (plus $PhCH_2OBpin$) into **4**-OTf (plus HBpin) - the 'reverse' reaction. (T = 298 K, t = 0 to 250 min).

(iv) Catalysis of the Tischenko reaction by systems of the type (Nacnac)^{Dipp}Al(X)(OCH_2Ph) in the absence of HBpin: To a solution of (Nacnac)^{Dipp}Al(X)(OCH_2Ph) (X = OTf, Et, H, OCH_2Ph) (0.021 mmol, 2 mol%) in benzene-d_6 (0.6 mL) was added benzaldehyde (107 µL, 1.05 mmol). The progress of the reaction was monitored by ^{1}H NMR spectroscopy (Figures S13 and S14).

.

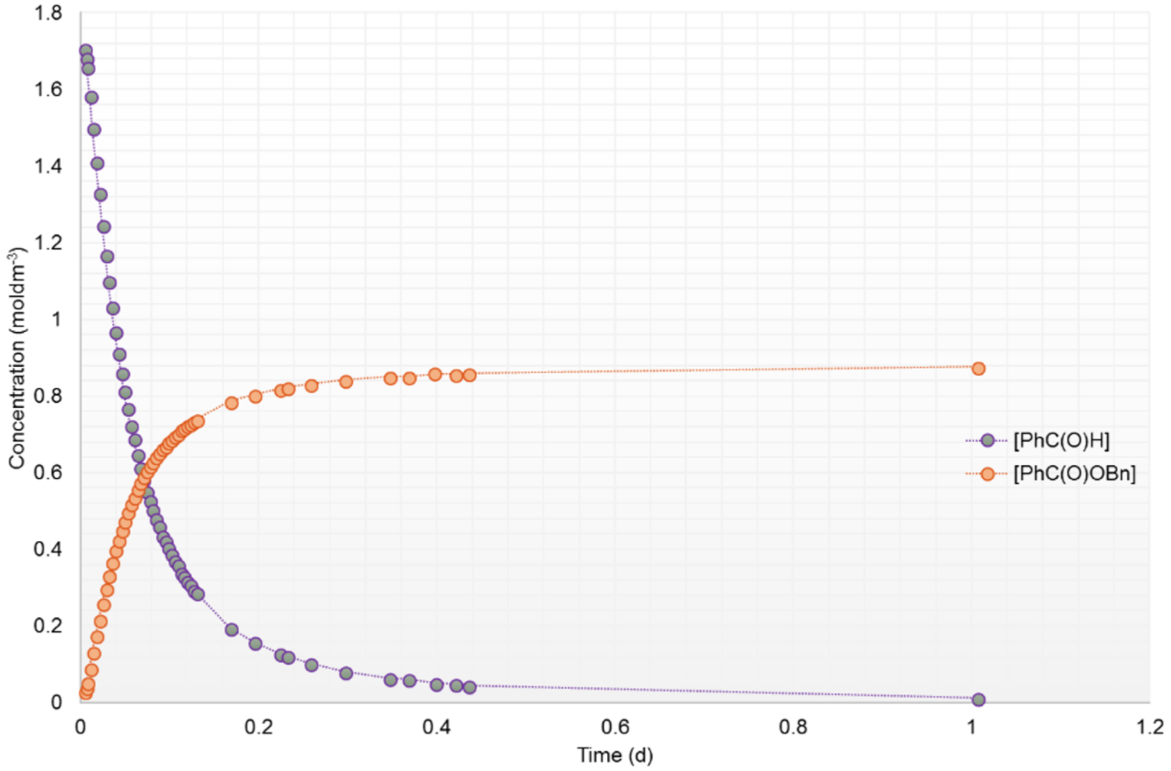

Figure S12: Temporal plot showing the formation of benzylbenzoate via the Tischenko disproportionation of benzaldehyde catalysed by **4**-OTf (T = 298 K, 2 mol%).

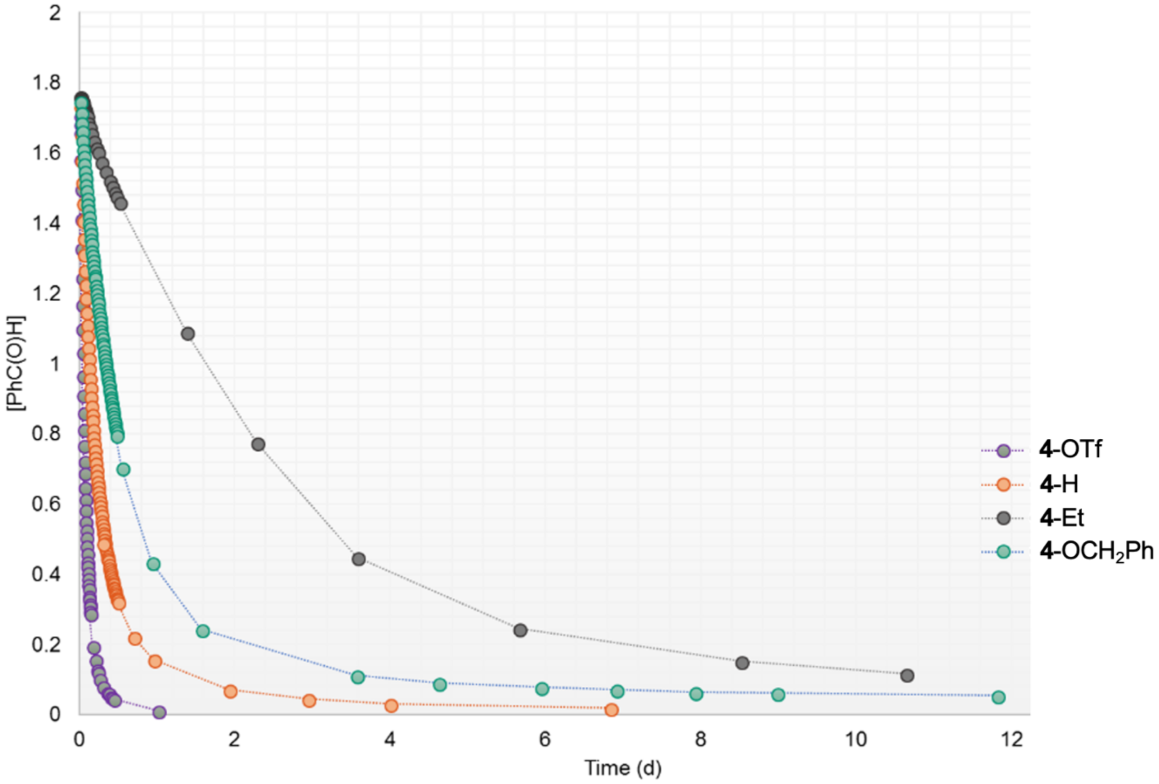

Figure S13: Comparative temporal plots showing the rate of consumption of benzaldehyde via the Tischenko reaction, catalysed by **4**-X (X = OTf, H, OCH_2Ph, Et; T = 298 K, 2 mol%). Turnover frequencies (TOFs, h^{-1}) calculated at 50% conversion: 2.08, 0.30, 0.10, 0.06, respectively.

(v) Background reaction between benzaldehyde and commercial HBpin

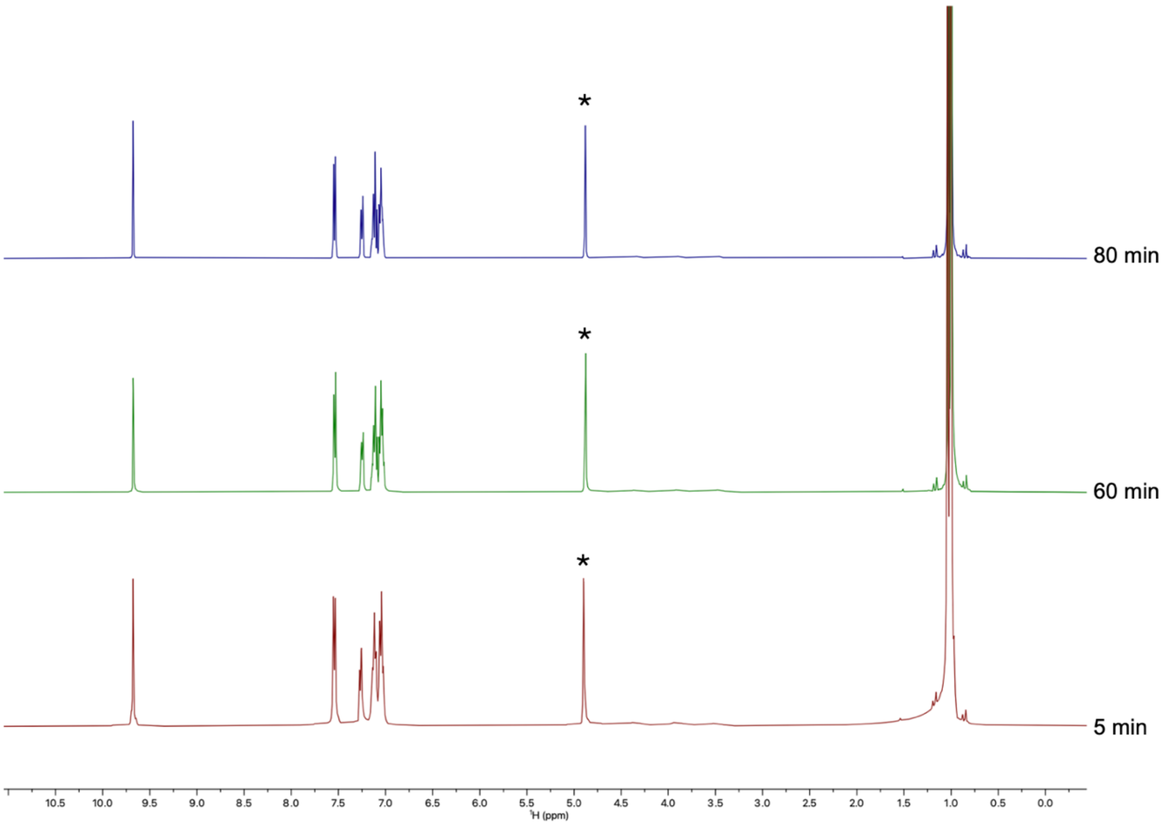

Figure S14: ^{1}H NMR spectra showing the background reaction between benzaldehyde and commercial HBpin (Aldrich). The resonance at 4.90 ppm marked * is due to the methylene protons of $PhCH_2OBpin$; integration against the aldehyde signal of benzaldehyde allows conversion to be estimated at 32% after 5 min, rising to 36% after 80 min. (Conditions: 2 mmol of each reagent in 1 mL of benzene-d_6 in a J. Young's NMR tube at room temperature).

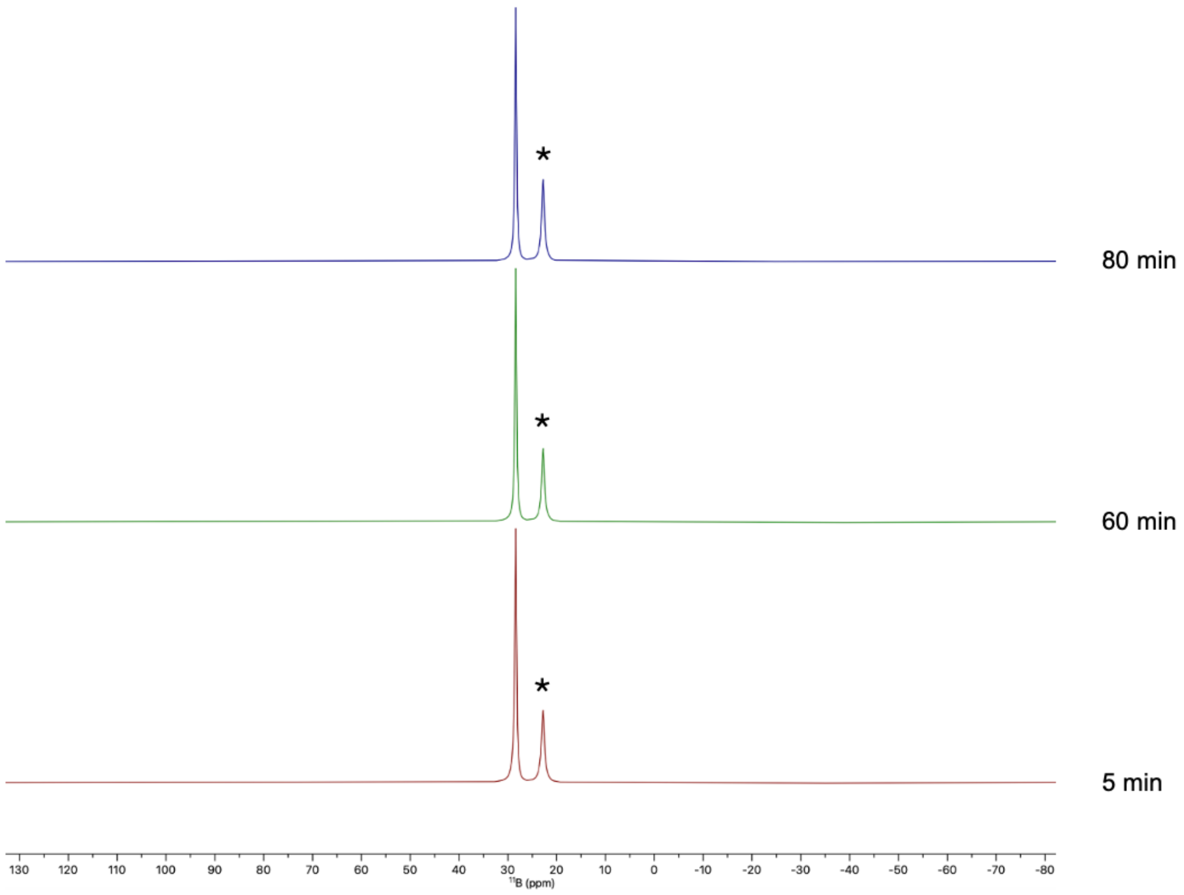

Figure S15: $^{11}B\{^{1}H\}$ NMR spectra showing the background reaction between benzaldehyde and commercial HBpin (Aldrich). The resonance marked * is due to $PhCH_2OBpin$. (Conditions: 2 mmol of each reagent in 1 mL of benzene-d_6 in a J. Young's NMR tube at room temperature).

(vi) Raw data/spectra for Figure 2 (hydroboration of acetophenone)

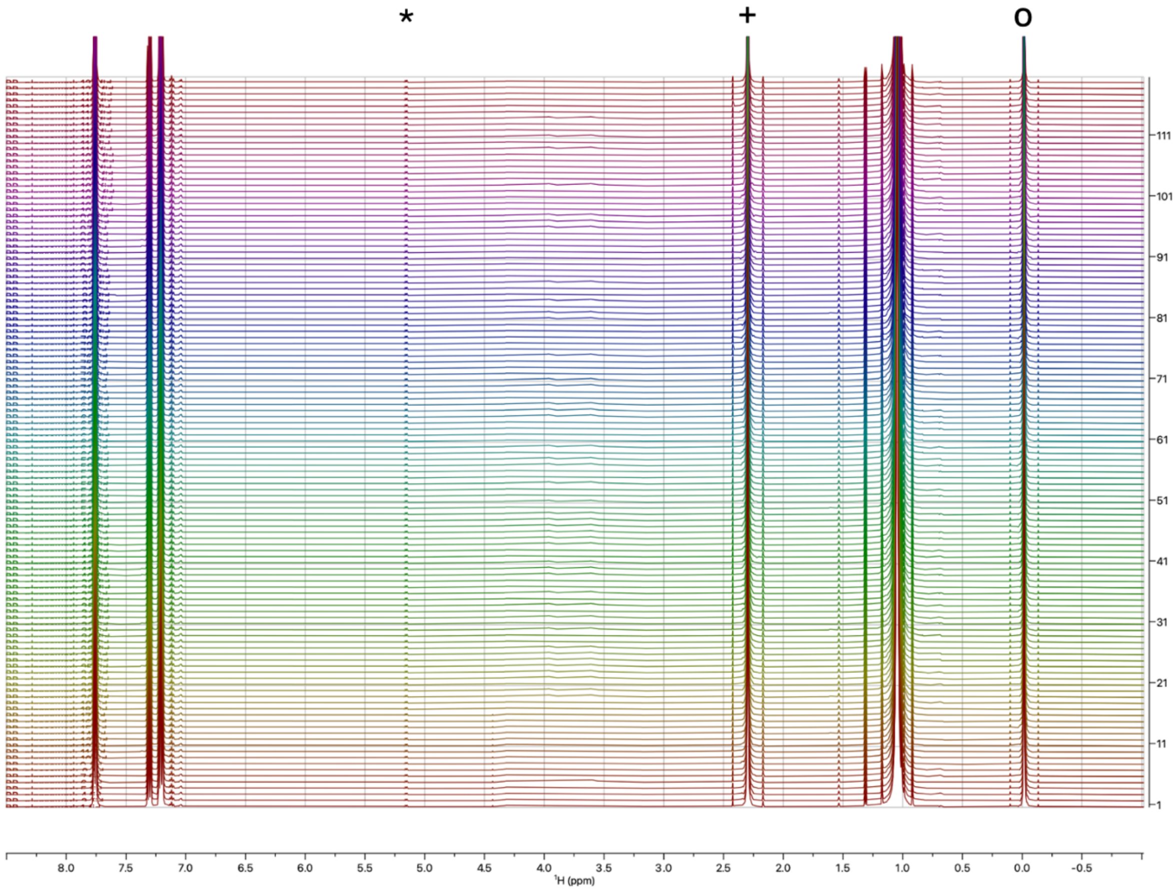

Figure S16: ^{1}H NMR spectra showing the progress of the reaction between acetophenone and *purified* HBpin at room temperature using $(Me_3Si)_2O$ as the internal standard. Spectra measured at 2 min intervals. Conversion (for main manuscript Figure 2) determined from the resonance due to PhC(Me)(H)OBpin at 5.3 ppm (*****) normalized to the internal standard (**o**) within the same spectrum (**+** = Me signal of acetophenone). (Conditions: acetophenone (0.24 mL, 2 mmol), HBpin (0.29 mL, 2 mmol), $(Me_3Si)_2O$ (27.5 mg, 0.17 mmol) in benzene-d_6 (0.1 mL)).

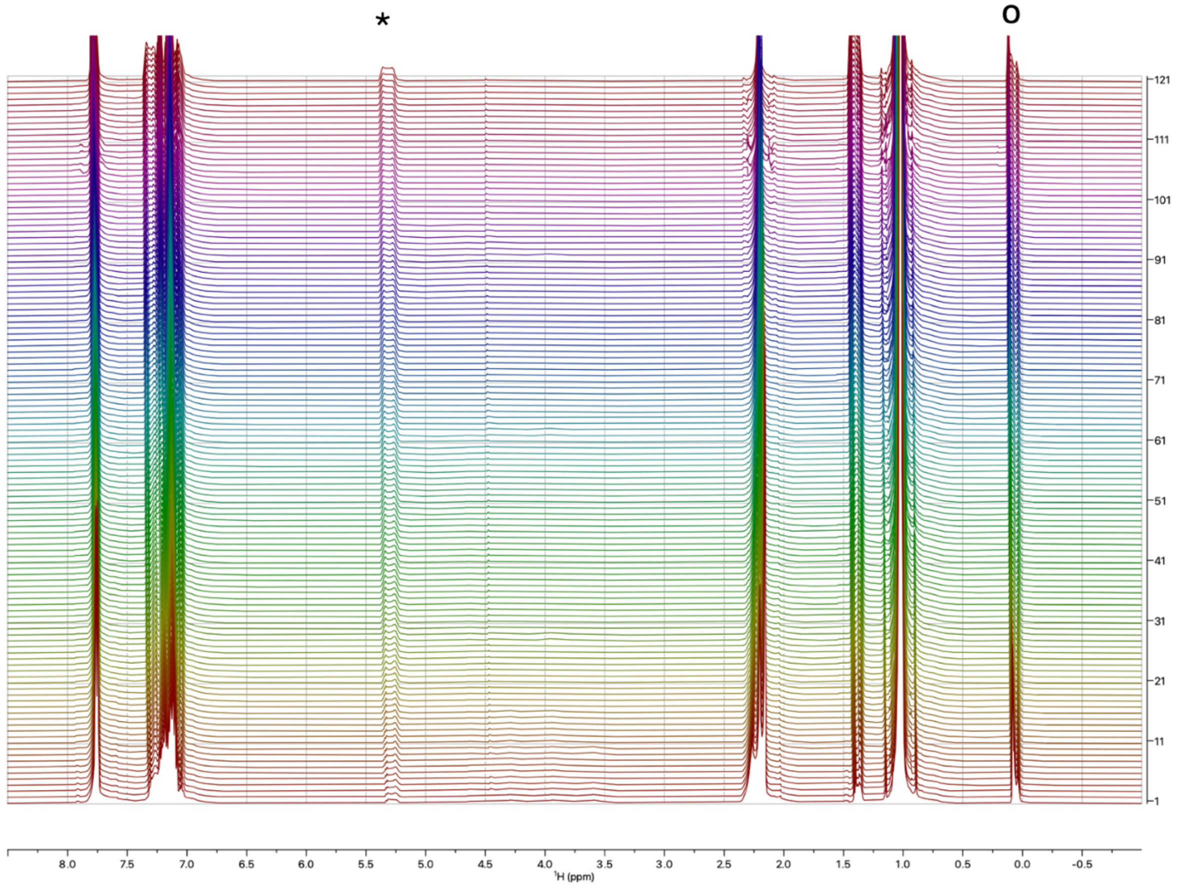

Figure S17: ^{1}H NMR spectra showing the progress of the reaction between acetophenone and *impure* (commercial/as received) HBpin at room temperature using $(Me_3Si)_2O$ as the internal standard. Spectra measured at 2 min intervals. Conversion (for main manuscript Figure 2) determined from the resonance due to PhC(Me)(H)OBpin at 5.3 ppm (*****) normalized to the internal standard (**o**) within the same spectrum. (Conditions: acetophenone (0.24 mL, 2 mmol), HBpin (0.29 mL, 2 mmol), $(Me_3Si)_2O$ (18.6 mg, 0.12 mmol) in benzene-d_6 (0.1 mL)).

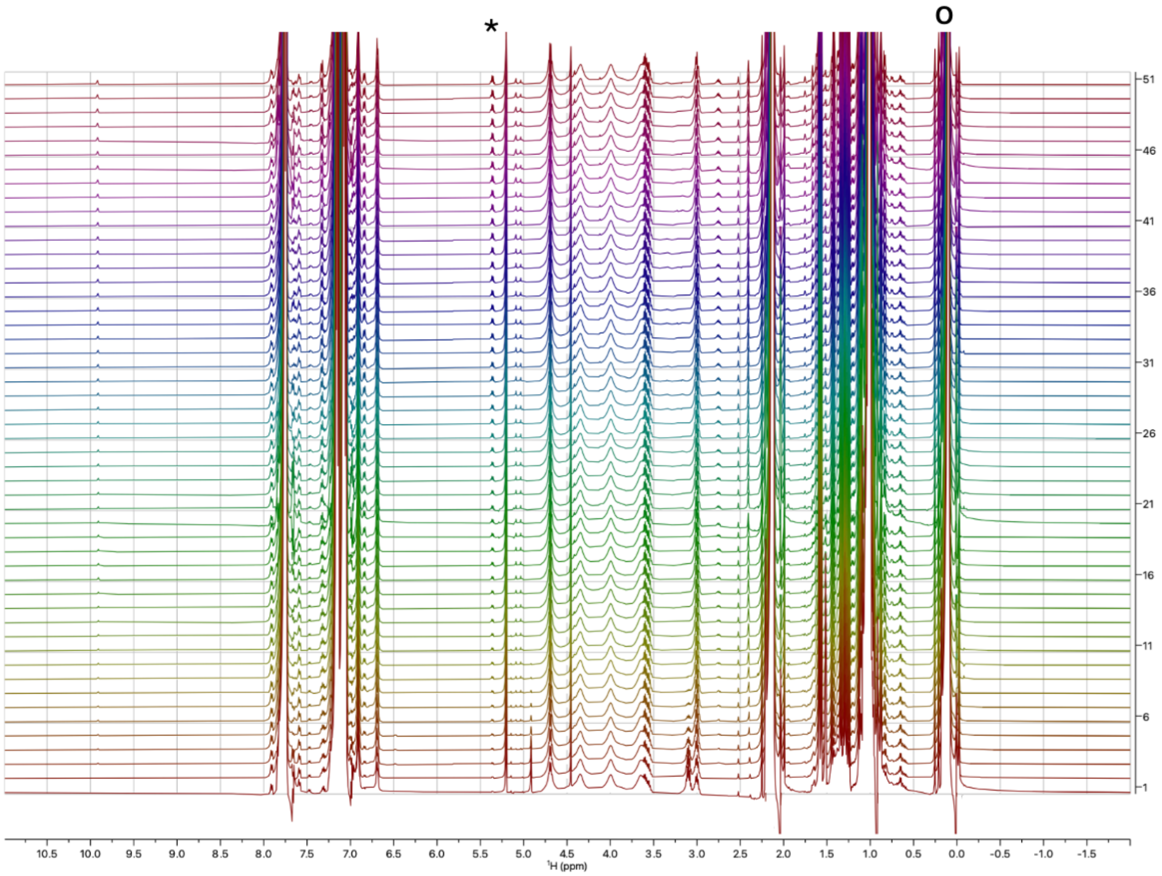

Figure S18: ^{1}H NMR spectra showing the progress of the reaction between acetophenone and *purified* HBpin in the presence of 2 mol% **3**-OTf at room temperature using $(Me_3Si)_2O$ as the internal standard. Spectra measured at 2 min intervals. Conversion (for main manuscript Figure 2) determined from the resonance due to PhC(Me)(H)OBpin at 5.3 ppm (*****) normalized to the internal standard (**o**) within the same spectrum. (Conditions: acetophenone (0.24 mL, 2 mmol), HBpin (0.29 mL, 2 mmol), **3**-OTf (23 mg, 0.04 mmol), $(Me_3Si)_2O$ (27.7 mg, 0.17 mmol) in benzene-d_6 (0.1 mL)).

(vii) NMR monitoring of reactions of HBpin with aluminium complexes 3-H, 3-OTf and 4-OTf

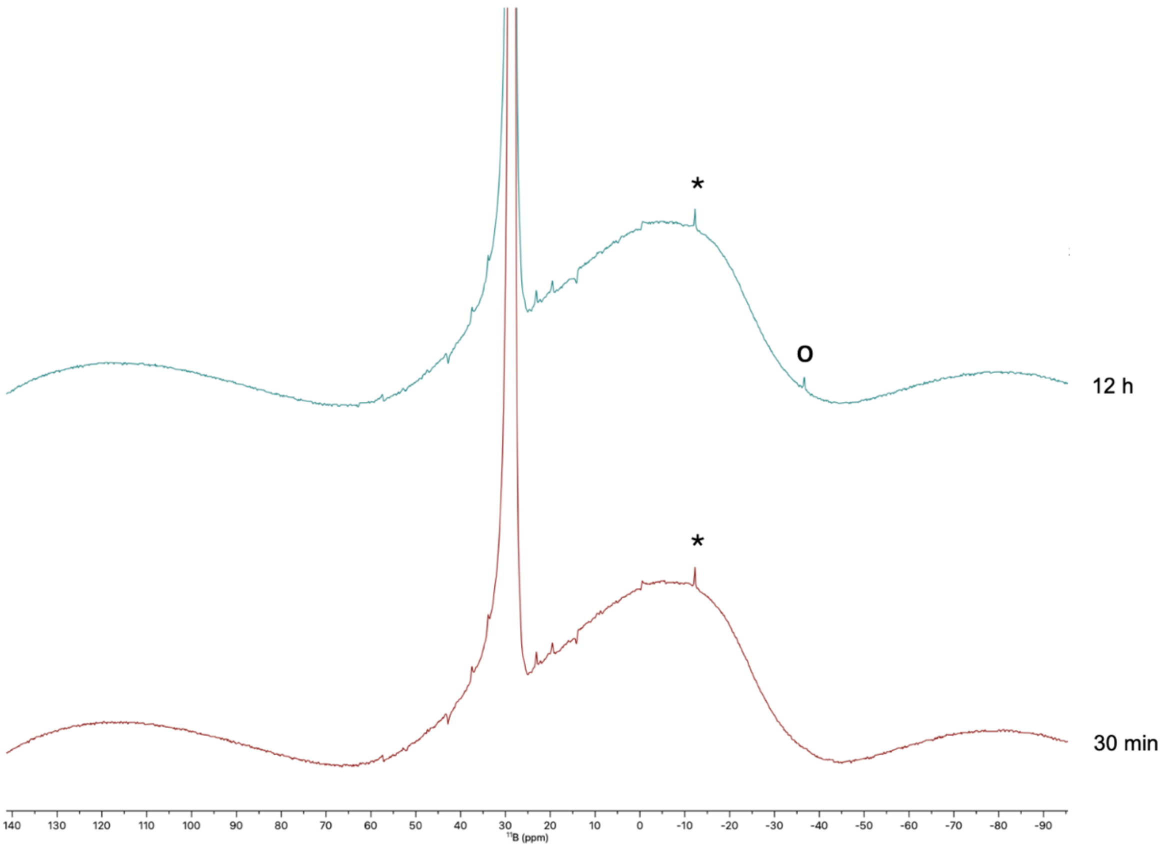

Figure S19: $^{11}B\{^{1}H\}$ NMR spectra showing the reaction of pre-purified HBpin with **3**-H (1 mol% **3**-H, benzene-d$_6$, room temperature). Signals identified: BH_3·L, 13.6 ppm (*****); (Nacnac)^{Dipp}Al(BH_4)$_2$, -37.0 ppm (**o**).

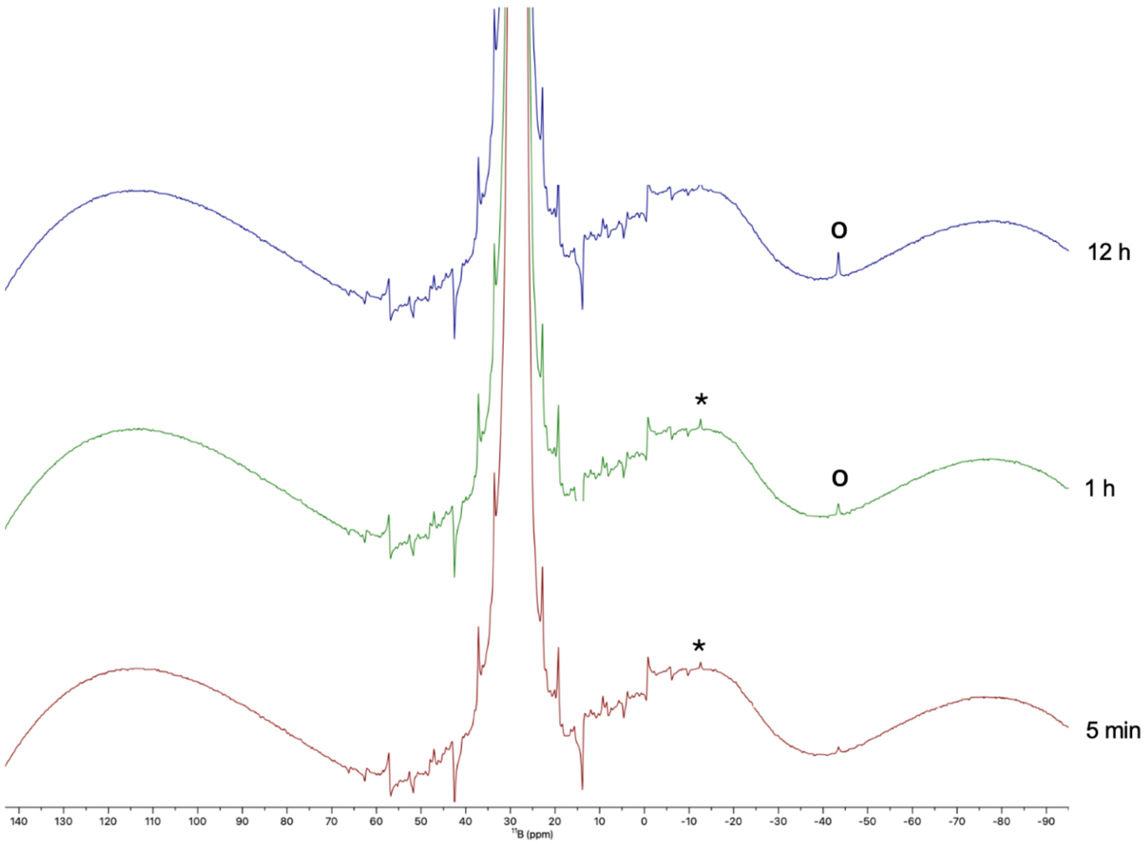

Figure S20: $^{11}B\{^{1}H\}$ NMR spectra showing the reaction of pre-purified HBpin with **3**-OTf (1 mol% **3**-OTf, benzene-d_6, room temperature). Signals identified: BH_3·L, 13.6 ppm (*); (Nacnac)^{Dipp}Al(OTf)(BH_4), -43.5 ppm (**o**).

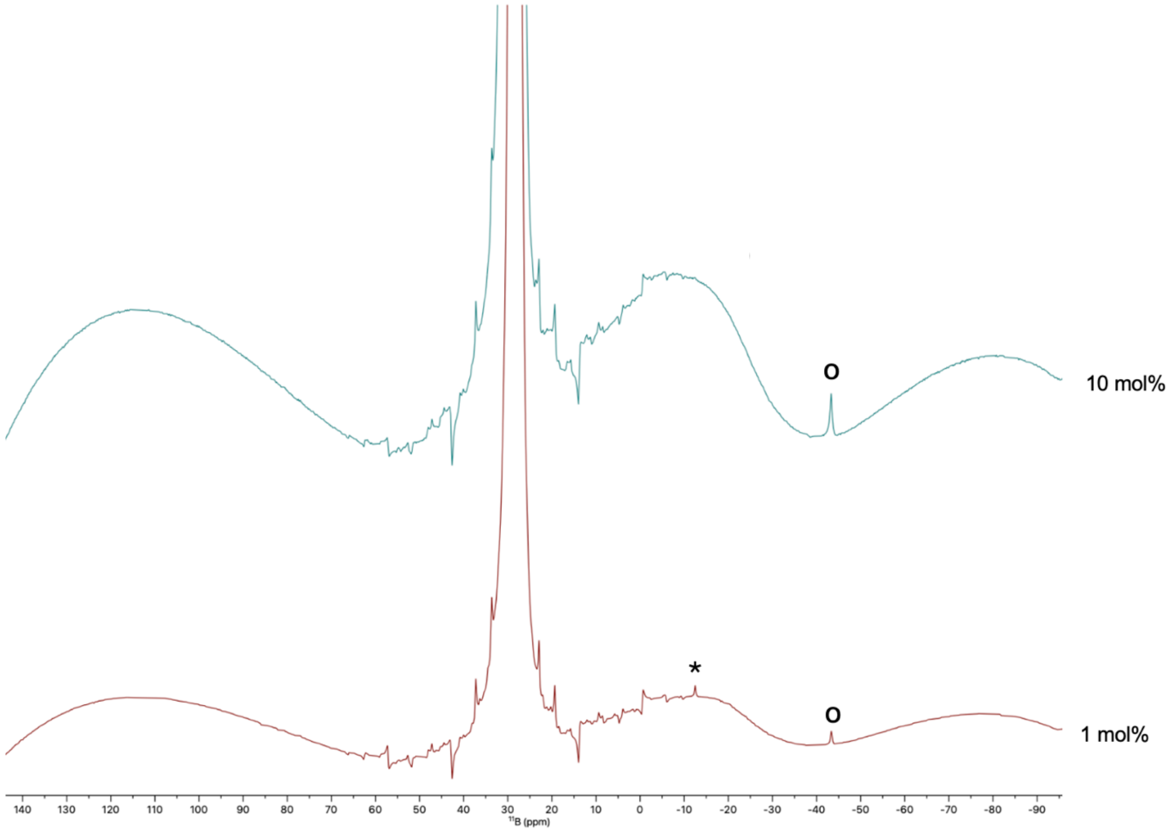

Figure S21: $^{11}B\{^1H\}$ NMR spectra showing the reaction of pre-purified HBpin with **3**-OTf at different concentrations of the alane (1 and 10 mol% **3**-OTf, benzene-d_6, room temperature, 16 h). Signals identified: $BH_3 \cdot L$, 13.6 ppm (*****); (Nacnac)^{Dipp}Al(OTf)(BH_4), -43.5 ppm (**o**).

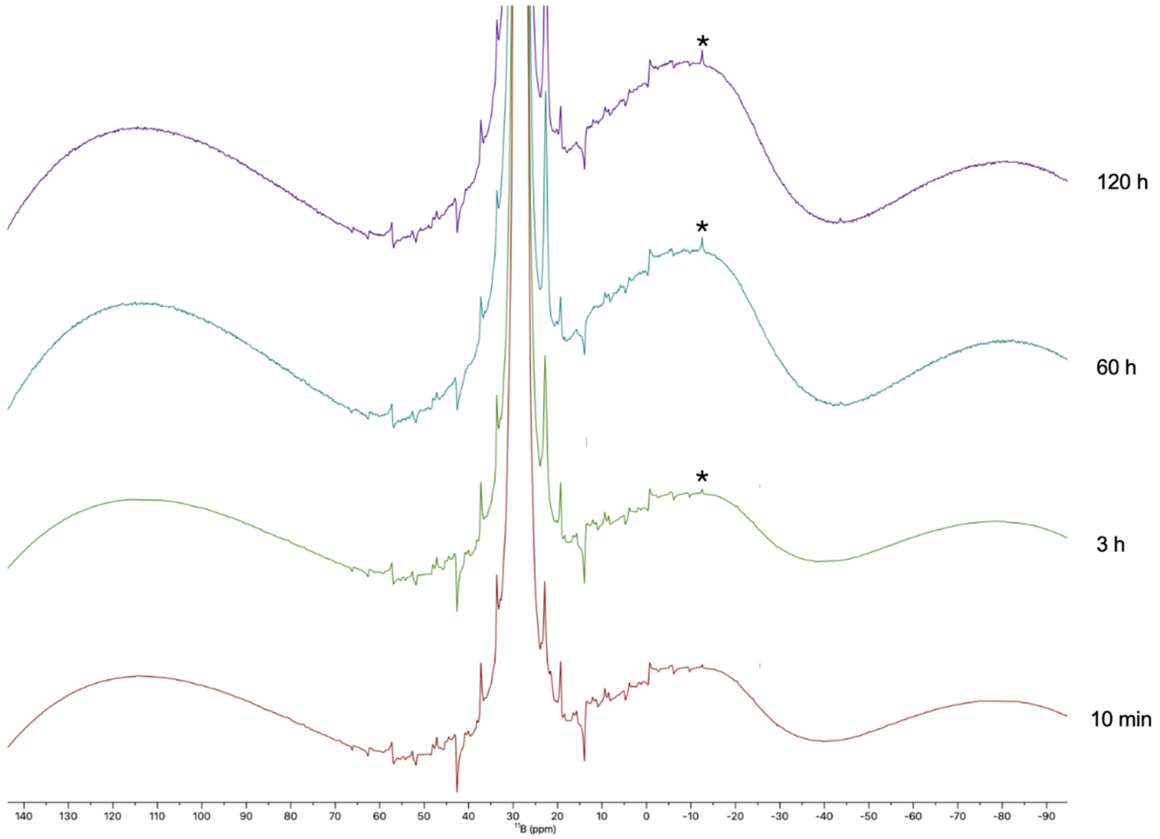

Figure S22: $^{11}B\{^{1}H\}$ NMR spectra showing the reaction of pre-purified HBpin with **4**-OTf (1 mol% **4**-OTf, benzene-d$_6$, room temperature). Signal identified: BH_3·L, 13.6 ppm (*****).

5. X-ray crystallography

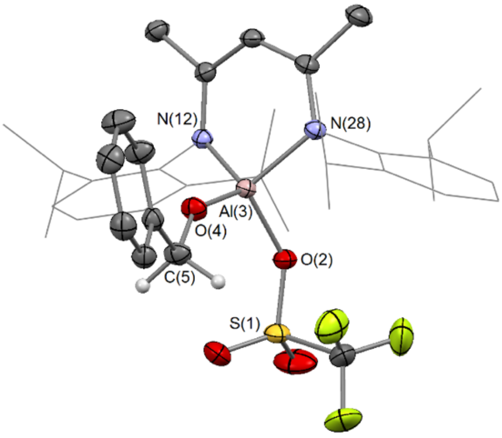

Figure S23: Molecular structure of **4**-OTf in the solid state as determined by X-ray crystallography. Most hydrogen atoms omitted and Dipp substituents shown in wireframe format for clarity; thermal ellipsoids set at the 40% probability level.

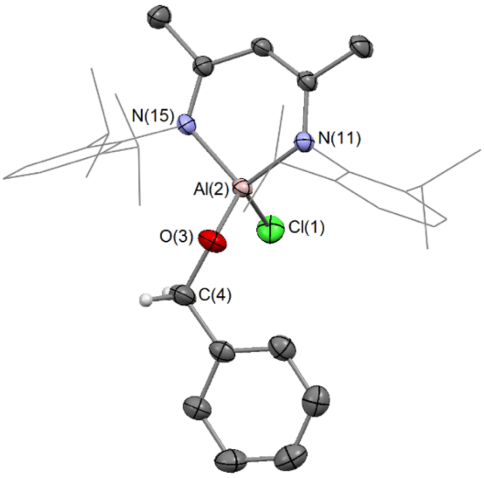

Figure S24: Molecular structure of **4**-Cl in the solid state as determined by X-ray crystallography. Most hydrogen atoms omitted and Dipp substituents shown in wireframe format for clarity; thermal ellipsoids set at the 40% probability level.

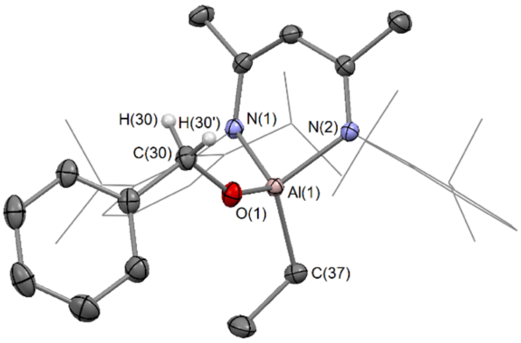

Figure S25: Molecular structure of **4**-Et in the solid state as determined by X-ray crystallography. Most hydrogen atoms omitted and Dipp substituents shown in wireframe format for clarity; thermal ellipsoids set at the 40% probability level.

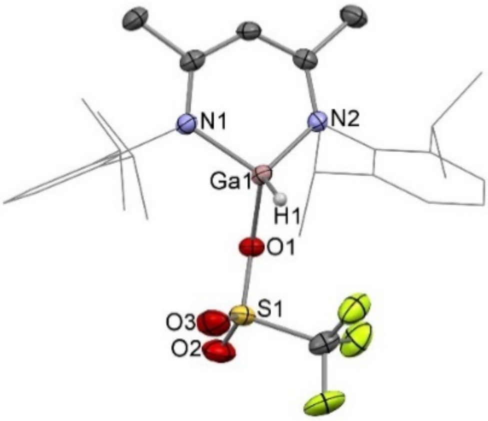

Figure S26: Molecular structure of (Nacnac)^{Dipp}Ga(H)(OTf) in the solid state as determined by X-ray crystallography. Most hydrogen atoms omitted and Dipp substituents shown in wireframe format for clarity; thermal ellipsoids set at the 40% probability level.

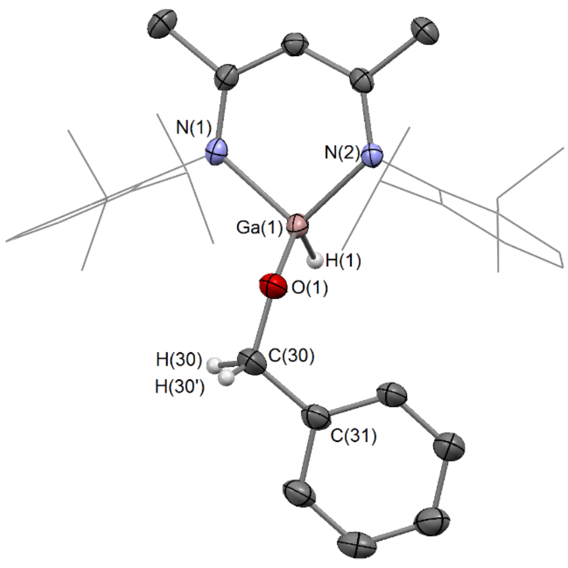

Figure S27: Molecular structure of (Nacnac)^{Dipp}Ga(H)(OCH_2Ph) in the solid state as determined by X-ray crystallography. Most hydrogen atoms omitted and Dipp substituents shown in wireframe format for clarity; thermal ellipsoids set at the 40% probability level.

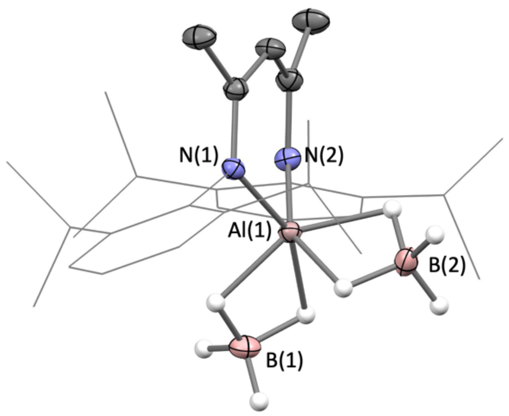

Figure S28: Molecular structure of (Nacnac)^{Dipp}Al(BH_4)$_2$ in the solid state as determined by X-ray crystallography. Most hydrogen atoms omitted and Dipp substituents shown in wireframe format for clarity; thermal ellipsoids set at the 40% probability level.

Table S1: X-ray crystallographic data collection and refinement parameters

	**4-OTf**	**4-Et**
**Formula**	$C_{37}H_{48}N_2AlF_3O_4S$	$C_{38}H_{53}N_2AlO$
**Fw (g mol^{-1})**	700.84	580.80
**Cell setting**	Monoclinic	Monoclinic
**Space group**	$P\ 2_1/n$	$P\ 2_1/c$
**a (Å)**	12.0514(1)	14.7787(3)
**b (Å)**	17.3947(2)	12.5452(3)
**c (Å)**	18.3356(2)	18.4876(5)
**α (°)**	90	90
**β (°)**	103.809(1)	91.020(2)
**γ (°)**	90	90
**V (Å^3)**	3732.61(7)	3427.09(14)
**Z**	4	4
**ρ_{calc} (g cm^{-3})**	1.247	1.126
**Radiation, λ (Å)**	1.54184	1.54184
**μ (mm^{-1})**	1.458	0.738
**Reflections collected**	22501	24207
**Independent reflections**	7739	7110
**$R_{(int)}$**	0.017	0.0284
**Parameters**	470	747
**R_1 (all data/I > 2σ(I))**	0.0368/0.0399	0.0376/0.0464
**ωR_2 (all data/I > 2σ(I))**	0.0941/0.0973	0.0964/0.1029
**T/K**	150(2)	150(2)
**CCDC Deposition No.**	2445261	2445258

Table S1 (contd.): X-ray crystallographic data collection and refinement parameters

	**4-Cl**	**(Nacnac)^{Dipp}Ga(OTf)H·C_7H_8**
**Formula**	$C_{36}H_{48}N_2AlClN_2O$	$C_{30}H_{42}N_2GaF_3O_3S{\cdot}C_7H_8$
**Fw (g mol^{-1})**	587.22	729.59
**Cell setting**	Monoclinic	Triclinic
**Space group**	*P* 2$_1$/*n*	*P*-1
**a (Å)**	9.5393(2)	10.4376(4)
**b (Å)**	17.3322(3)	12.2280(4)
**c (Å)**	20.7329(3)	17.2794(5)
**α (°)**	90	70.314(3)
**β (°)**	93.031(2)	81.733(3)
**γ (°)**	90	79.506(3)
**V (Å^3)**	3423.12(11)	2033.65(13)
**Z**	4	2
**ρ_{calc} (g cm^{-3})**	1.139	1.191
**Radiation, λ (Å)**	1.54180	1.54180
**μ (mm^{-1})**	1.446	1.797
**Reflections collected**	19072	8405
**Independent reflections**	7025	8373
**$R_{(int)}$**	0.036	0.020
**Parameters**	370	424
**R_1 (all data/I > 2σ(I))**	0.0435/0.0510	0.0506/0.0471
**ωR_2 (all data/I > 2σ(I))**	0.1104/0.1169	0.1204/0.1173
**T/K**	150(2)	150(2)
**CCDC Deposition No.**	2445259	2445257

Table S1 (contd.): X-ray crystallographic data collection and refinement parameters

	**(Nacnac)^{Dipp}Ga(H)(OCH$_2$Ph)**	**(Nacnac)^{Dipp}Al(BH$_4$)$_2$**
**Formula**	$C_{36}H_{49}N_2GaO$	$C_{29}H_{49}N_2AlB_2$
**Fw (g mol^{-1})**	595.49	474.30
**Cell setting**	Monoclinic	Monoclinic
**Space group**	$P\ 2_1/c$	$P\ 2_1/c$
**a (Å)**	18.3437(5)	18.0017(2)
**b (Å)**	9.0495(2)	9.9457(1)
**c (Å)**	40.5307(9)	17.5909(1)
**α (°)**	90	90
**β (°)**	99.065(2)	109.737(1)
**γ (°)**	90	90
**V (Å^3)**	6644.1(3)	2964.44(5)
**Z**	8	4
**ρ_{calc} (g cm^{-3})**	1.191	1.063
**Radiation, λ (Å)**	1.54184	1.54184
**μ (mm^{-1})**	1.338	0.714
**Reflections collected**	39664	45704
**Independent reflections**	13794	6131
**$R_{(int)}$**	0.0356	0.0245
**Parameters**	747	349
**R_1 (all data/I > 2σ(I))**	0.0618/0.0409	0.0341/0.0364
**ωR_2 (all data/I > 2σ(I))**	0.1134/0.0984	0.089/0.090
**T/K**	150(2)	100(2)
**CCDC Deposition No.**	2445260	2445262

6. DFT calculations on the inter-conversion of 3-OTf and 4-OTf

The methodology used to probe the mechanism linking **3**-OTf and **4**-OTf involved the BP86-D3 functional with 6-31G(d) for Al, S, O, N, C from the carbonyl-derived fragment and for B- and Al-bound H, and 6-31G for other C and H; derived energies were corrected with single point calculations at the B3LYP-D3/ 6-311G** level of theory (overall: B3LYP-D3/6-311G**//BP86-D3/BS1). All intermediates were verified as local minima by frequency calculations. Transition States (TS) structures were confirmed as such through the presence of a single imaginary frequency and by intrinsic reaction coordinate.

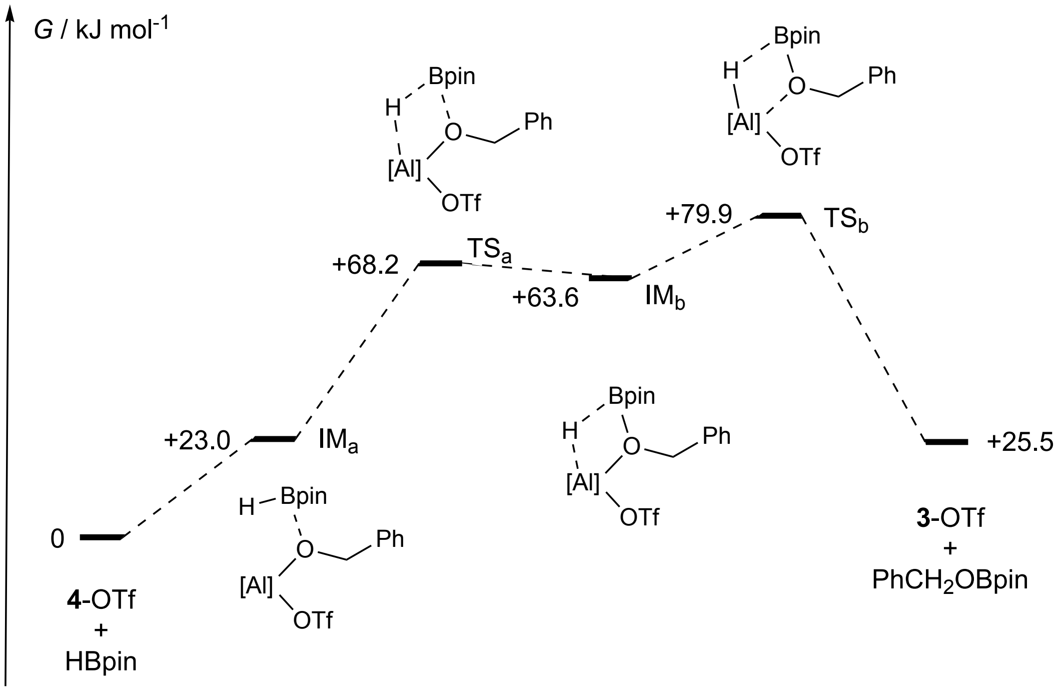

Figure S29: Calculated mechanism for the inter-conversion of **3**-OTf/$PhCH_2OBpin$ and **4**-OTf/HBpin (energies in kJ mol^{-1}; [Al] = {HC(MeCDippN)$_2$}Al).

7. References for supporting information

[S1] Z. Yang, M. Zhong, X. Ma, S. De, C. Anusha, P. Parameswaran, H. W. Roesky, *Angew. Chem. Int. Ed*. **2015**, *54*, 10225–10229.

[S2] A. Caise, D. Jones, E. L. Kolychev, J. Hicks, J. M. Goicoechea, S. Aldridge, *Chem.-Eur. J.* **2018**, *24*, 13624-13635.

[S3] S. Singh, H.-J. Ahn, A. Stasch, V. Jancik, H. W. Roesky, A. Pal, M. Biadene, R. Herbst-Irmer, M. Noltemeyer, H.-G. Schmidt, *Inorg. Chem.* **2006**, *45*, 1853–1860.

[S4] L. K. Keyes, A. D. K. Todd, N. A. Giffin, A. J. Veinot, A. D. Hendsbee, K. N. Robertson, S. J. Geier, J. D. Masuda, *RSC Adv.* **2017**, *7*, 37315–37323.

[S5] S. Harder, J. Spielmann, *Chem. Commun.* **2011**, *47*, 11945–11947.

[S6] G. C. Lloyd-Jones, N. P. Taylor, *Chem.-Eur. J.* **2015**, *21*, 5423–5428; (b) R. Kumar, R. K. Meher, J. Sharma, A. Sau, T. K. Panda, *Org. Lett.* **2023**, *25*, 7923–7927.
